# Multi-Omics Profiling of Long Noncoding RNAs in Clear Cell Renal Cell Carcinoma for Characterization and Clinical Applications

**DOI:** 10.7150/ijbs.127291

**Published:** 2026-03-25

**Authors:** Yuhong Ding, Yang Li, Zhenghao Liu, Yaxin Hou, Zhipeng Yao, Pengjie Shi, Jinxu Li, Yingchun Kuang, Yiting Liu, Junyi Hu, Lilong Liu, Ke Chen

**Affiliations:** 1 Department of Urology, Tongji Hospital, Tongji Medical College, Huazhong University of Science and Technology, Wuhan, China.; 2 Institute of Urology, Tongji Hospital, Tongji Medical College, Huazhong University of Science and Technology, Wuhan, China.

**Keywords:** ccRCC, long non-coding RNA, multi-omics, biomarkers, diagnosis, prognosis

## Abstract

Clear cell renal cell carcinoma (ccRCC), the most common and lethal subtype of renal cell carcinoma, exhibits marked intratumoral heterogeneity and complicates clinical management. Although long noncoding RNAs (lncRNAs) regulate diverse cellular processes, their landscape and biomarker potential in ccRCC remain poorly defined. Here we performed single-nucleus and bulk transcriptomic, proteomic, and metabolomic analyses on a cohort of 100 ccRCC patients. The expression pattern of lncRNAs were described based on metacells. Malignant cells displayed broader but lower lncRNA expression, likely reflecting copy number alterations, whereas low-abundance lncRNAs in normal epithelial cells showed individual variability. Multi-omics integration was used to establish a preliminary lncRNA functional inference pipeline, identifying lncRNAs involved in metabolic and immune processes and validating their roles through functional and *in vivo* experiments. Candidate biomarkers lncRNAs were identified to build diagnostic (DMRlnc) and prognostic models (PMRlnc), which were validated in TCGA, CheckMate, and IMmotion151 cohorts. DMRlnc achieved high diagnostic accuracy in both discovery and TCGA-KIRC cohorts (AUC 0.98 and 0.93). PMRlnc stratified patients into distinct risk groups with significant differences (p < 0.0001) across TCGA-KIRC and IMmotion151 cohorts. PMRlnc further indicated that low-risk patients may benefit more from nivolumab, while high-risk patients might respond better to atezolizumab plus bevacizumab.

## Introduction

Renal cell carcinoma (RCC) is among the ten most common malignancies worldwide, with an increasing incidence but a declining mortality rate 1,2. In 2024, RCC ranked fifth among men (5%) and ninth among women (3%) in the United States 1. As the predominant subtype, ccRCC accounts for death rate 3, which highlighting the need for deeper insight into its development and progression. However, the significant intratumoral heterogeneity (ITH) in ccRCC leads to variable clinical outcomes and complicates management 4, underscoring the need for precision prognostic strategies. Given its hallmarks of metabolic dysregulation 5 and responsiveness to immunotherapy 6, identifying potential regulators of the metabolic and immunological mechanisms of ccRCC is critical for elucidating ITH. Besides, the renal mass biopsy remains the diagnostic gold standard, but its invasiveness underscores the value of less invasive approaches such as liquid biopsy. Therefore, these challenges point to the urgent need for identifying informative regulators and developing clinically applicable strategies for diagnosis, prognosis, and precision therapy in ccRCC.

Defined as transcripts longer than 200 nucleotides without protein-coding potential 7, lncRNAs show distinct expression patterns in tumors compared with normal tissues, yet their global landscape in ccRCC remains largely unexplored. LncRNAs play critical regulatory roles in cancer biology, including posttranslational modifications and epithelial-mesenchymal transitions 8,9. However, how lncRNAs contribute to ccRCC progression through metabolic or immunological pathways is still not well understood 10. In particular, the systematic functions and underlying mechanisms by which lncRNAs regulate metabolic and immunological processes remain to be elucidated. Moreover, with their stability and detectability in body fluids, lncRNAs are emerging as promising candidates for liquid biopsy 11, suggesting their potential utility as diagnostic and prognostic biomarkers to support clinical decisions.

In our study, we collected a large cohort of 100 ccRCC samples and 50 corresponding normal adjacent tissues (NATs). Single-nuclei sequencing (snRNA-seq), bulk RNA-seq, proteomics, and untargeted metabolomics were integrated to characterize lncRNA expression patterns between tumor and normal nuclei. We further established a pipeline for functional annotation of lncRNAs, with a focus on metabolic and immunological pathways and validated by functional experiments. In addition, we constructed diagnostic and prognostic models based on lncRNAs, which were validated in large external ccRCC cohorts (TCGA-KIRC, CheckMate, IMMOTION151) and demonstrated strong performance.

## Material and Methods

### Cohorts

**TJ-RCC cohort**. The in-house cohort was described as previous study 12, while clinical and pathological information has been listed as Table S1.

**TCGA-KIRC cohort**. Normalized expression matrices and paired clinical data were directly retrieved from the Genomic Data Commons Data Portal 13 and UCSC Xena 14, encompassing 533 ccRCC samples and 72 matched NATs.

**IMMOTION151 cohort**. It was a phase 3 trial that compared atezolizumab plus bevacizumab with sunitinib in untreated RCC patients 15. A total of 823 fastq data cases were obtained from the European Genome-phenome Archive with permission.

**CheckMate cohort.** Merged by CheckMate 025 cohort (phase III) with the CheckMate 009 cohort (phase I) and the CheckMate 010 cohort (phase II) 16, clinical data of 311 RCC patients comparing nivolumab with everolimus in patients with RCC who had previously been treated with one or two anti-angiogenic regimens were included.

### Statistical analysis

All statistical analyses of sequencing in this study were conducted using R software (v4.3.2) and python (v3.8) on Windows and Linux system. Two-sided tests were used for all analyses, with a P-value < 0.05 considered statistically significant. Experimental data were presented as mean ± standard deviation from at least three independent experiments. Comparisons between two groups were performed using two-tailed Student's t-test, while comparisons among multiple groups were conducted using one-way analysis of variance (ANOVA). Repeated measurements over time were analyzed using two-way ANOVA.

### Single-nucleic transcriptome

**Processing of snRNA-seq.** Upstream data processing was performed as described previously 17. In Seurat workflow (v4.3.0) 18, cells with < 1000 UMIs or > 10% mitochondrial transcripts were removed as low-quality, yielding 94703 nuclei after quality control. The data were then normalized and standardized, with 3000 variable genes selected for downstream analysis, followed by dimension reduction using principal component analysis (PCA; 50 components). Batch effects were corrected with harmony (v1.2.0) 19, and clusters were identified with FindNeighbors and FindClusters (resolution = 2.3). Major cell types were annotated based on established protein markers (listed on Table S2) and visualized by uniform manifold approximation and projection (UMAP). NAT samples were processed using the same workflow, and proximal tubule (PT) nuclei were integrated with malignant nuclei to generate a combined dataset.

**Cluster accuracy.** LncRNAs with cell type specific expression were identified from 10 samples and their ability to discriminate subclusters was evaluated in other 10 samples (Table S2). Using the selected lncRNAs or classic markers as features, dimension reduction and clustering were performed following the pipeline described above. Clustering quality based on intrinsic features (coordinates and distances) was evaluated using the silhouette coefficient (cluster, v2.1.6 20), Davies-Bouldin index (fpc, v2.2.13 21), and Calinski-Harabasz index (clusterSim, v0.51.5 22). External validation was performed using the adjusted rand index (ARI) and normalized mutual information (NMI) to quantify the concordance between the re-clustered results and reference annotations, as implemented in the mclust (v6.1.1 23) and aricode (v1.0.3) 24 packages.

**Expression analysis of lncRNAs.** To reduce data sparsity while preserving cellular heterogeneity, nuclei with highly similar expression profiles were aggregated into units termed metacells for subsequent analyses using the MetacellsByGroups function in the hdWGCNA package (v0.3.1) 25. By default, 1000 metacells per cell type were generated to construct a matrix, which was subsequently used to analyze lncRNA expression patterns.

**Copy number analysis.** Copy number variations (CNVs) in malignant and PT nuclei were estimated using inferCNV (v1.18.1) 26, with PT nuclei serving as the baseline reference. The raw count matrix was extracted from the seurat object, with the denoise parameter set to True. CNV scores were retrieved from the 'inferCNV. Observations' file generated during the infercnv::run procedure.

**hdWGCNA analysis** 25**.** Each major cell type was extracted as an individual seurat object for high-dimension weighted correlation network analysis (hdWGCNA), a biology approach used to characterize gene association patterns across samples. To mitigate data sparsity, transcriptionally similar nuclei were aggregated into metacells using the MetacellsByGroups function with the k-nearest neighbors' algorithm (*group.by* = “major cell type”, *k* = 20, with other default settings), resulting in a metacell expression matrix. After normalization and standardization, a soft-thresholding power was automatically selected using default parameters to construct a co-expression network with scale-free topology and modular structure. Modules were identified by unsupervised clustering using the dynamic tree-cutting algorithm with default settings. Hub genes within each module were determined by calculating module eigengenes and ranking genes according to eigengene connectivity (kME) values derived from the ModuleEigengenes and ModuleConnectivity function. UMAP was then applied to visualized the hdWGCNA network into a low-dimensional network.

### Bulk transcriptome

**Data process.** Using default parameters on a Linux system, raw FASTQ files underwent quality control, including adapter clipping, quality filtering, and pre-read quality clipping by fastp (v0.24.0) 27. The cleaned paired-end reads were aligned to the UCSC hg38 reference genome which including the non-coding transcripts using HISAT2 (v1.18.1) 28 to generate SAM files. SAM files were subsequently converted to BAM format, followed by sorted and indexed using samtools (v1.21) 29 before import into R. Gene-level read counts were obtained with FeatureCounts function in the Rsubread package (v2.16.1) 30. Batch effects were estimated and corrected using the sva package (v3.50.0) 31. The raw counts were converted into transcripts per million (TPM) for differential expression and correlation analyses. This process generated an expression matrix comprising 10890 lncRNAs and 18181 protein-coding transcripts.

**Enrichment analysis.** The gene ontology enrichment (GO) enrichments and Kyoto Encyclopedia of Genes and Genomes (KEGG) enrichments was conducted by the clusterProfiler package (v4.10.1) 32. The gene set enrichment analysis (GSEA) was performed by fgsea package (v1.32.4) 33.

**Correlation analysis.** The TPM, metabolome, and proteome matrices were scaled prior to correlation analysis. The correlation matrix of target lncRNAs was derived for data filtering. Only lncRNA-metabolite or lncRNA-protein pairs with statistically significant P values were retained calculated by corPvalueStudent in hdWGCNA and visualized using pheatmap (v1.0.12) 34. The heatmaps displaying pairs with correlation coefficients greater than 0.3 was generated.

**Differential expression lncRNAs analysis.** Differentially expressed lncRNAs (DElncRNAs) were identified from TPM matrices using Wilcoxon tests between 100 ccRCC tissues and 50 matched NATs (set1). LncRNAs with |log2FC| > 1 and adjusted P < 0.05 were defined as significant. A similar approach was applied to identify DElncRNAs among the four ccRCC subtypes (set2). Visualization was performed using the R package pheatmap.

**Clustering.** LncRNA markers were identified across 12 subtypes using the FindAllMarkers function in Seurat package, and utilized to classify the subtypes of ccRCC. Consensus clustering analysis was performed using the ConsensusClusterPlus package (v1.66.0) 35 with the partitioning around medoids algorithm and pearson distance. The optimal number of clusters was determined based on the cumulative distribution function (CDF), with the delta area plateauing at k = 4, indicating limited gains in clustering stability with higher k values (Figure S2C). Additionally, non-negative matrix factorization (NMF) clustering (v0.27) was conducted with default parameters 36, and the factorization rank was selected according to the cophenetic correlation coefficient, which showed a marked decrease at k = 4, suggesting reduced clustering robustness beyond this point (Figure S2D). Accordingly, k = 4 was selected as the optimal number of clusters for downstream analyses.

### Untargeted metabolomics and proteomics

**Data process.** The main process and parameters were set according to a previous study 12. The output matrix was normalized for downstream analysis.

**Metabolism enrichment analysis.** The target metabolites clustered in each heatmap were subjected to pathway enrichment using MetaboAnalyst6.0 37. Compound names were input as a list for pathway analysis based on the SMPDB metabolite set library 38. The super-class of metabolite were indexed from the Human Metabolome Database 39.

### Model building and testing

**Model building.** The TPM matrix was centralized using preProcess in caret package (v6.0-94) 40, and subsequently randomly divided into independent training (75%) and testing (25%) sets. For DMRlnc, features were defined as the intersection of lncRNA markers, DElncRNAs (set1), and lncRNAs detected in exoRBase 41. For PMRlnc, features were derived from the overlap between DElncRNAs (set2) and lncRNA markers. To prevent overfitting, recursive feature elimination (RFE) was applied as a wrapper-based feature selection method. RFE with 10-fold cross validation was repeated 100 times. The five lncRNAs with the highest selection frequencies were retained to construct PMRlnc and only statistically significant lncRNAs were included. Both DMRlnc and PMRlnc were built using logistic regression in independent training sets, with thresholds optimized by Youden's J statistic 42.

**Model testing.** DMRlnc was applied to the scaled in-house testing sets and the TCGA-KIRC cohort. Model performance was evaluated using receiver operating characteristic (ROC) curves and area under the curve (AUC) values with 95% confidence intervals, calculated with the pROC package (v1.18.5) 43. Sensitivity, specificity, and confusion matrices were calculated to summarize the discriminatory ability. Similarly, PMRlnc was validated in the in-house validation set and further applied to the scaled TCGA-KIRC, CheckMate, and IMMOTION151 cohorts. Multivariable Cox regression was performed using the coxph function and visualized using the ggforest function in the survival package (v3.5-7) 44 to assess the independent prognostic value of PMRlnc. Time-dependent ROC, Survival analyses and adjusted Kaplan-Meier (KM) curves were conducted to evaluate risk stratification by PMRlnc using the survminer (v0.4.9) 45 and adjustedCurves (v0.1.3) 46. In the TCGA-KIRC cohort, prognostic accuracy was further evaluated using time-dependent ROC analysis with the timeROC package (v0.4) 47, while net reclassification improvement (NRI) and integrated discrimination improvement (IDI) were calculated using the survIDINRI (v1.1.2) 48 packages. The calibrate curve and time-dependence barrier score was calculated by rms package (v8.1.0) 49 and pec package (v2025.6.24) 50, and decision curve analysis (DCA) was performed using the ggDCA package (v1.2) 51.

### Experiments

**Cell lines.** The 786O and 769P cell lines were obtained from SUNNCELL with STR profiling. 786O cells were cultured in Dulbecco's modified eagle medium (DMEM; Gibco) supplemented with 10% fetal bovine serum (Gibco), 100U/mL penicillin, and 100µg/mL streptomycin (Servicebio, catalog #G4003), whereas 769P cells were maintained in Roswell-park memorial institute 1640 medium (RPMI-1640; Gibco) with the same supplements. Following transduction with overexpressing or control lentiviruses (Corues Biotechnology), cells were selected with puromycin (BioFroxx, catalog #1299MG025) for three days (4µg/mL for 786O and 2µg/mL for 769P) and subsequently maintained in medium containing puromycin at half the selection concentration.

**Citric acid measurement.** Citric acid levels were measured using the Amplex Red assay (Beyotime, S0335S). Cells were lysed in 100-200μL lysis buffer per 1×10⁶ cells on ice, centrifuged at 12000g at 4 °C, and supernatants were collected. Samples or standards (20μL) were mixed with 80μl reaction solution in black, clear-bottom 96-well plates and incubated at 37 °C for 60 min in the dark. Fluorescence was measured at 560/590nm, citrate concentrations were calculated from a standard curve, and values were normalized per mg protein.

**ATP measurement.** Adherent cells were lysed in ice-cold lysis buffer and centrifuged at 12000g at 4 °C to collect the supernatant (Beyotime, catalog #S0026). A series of ATP standards was prepared to generate a standard curve. Subsequently, 100 µL of working solution was added to each well in white 96-well plates and incubated at room temperature for 5min, followed by the addition of 20 µL of sample or standard. The reaction mixture was immediately mixed, and relative luminescence units (RLU) were measured using a luminometer. ATP concentrations were calculated from the standard curve and normalized to protein content, with results expressed as nmol ATP per mg protein.

**Oxygen consumption rate (OCR) and Extracellular acidification rate (ECAR).** Cells were seeded at a density of 1×10^5^ cells per well in black, clear-bottom 96-well plates. Working solutions and oligomycin (MCE, catalog #HY-N6782) were prepared and added according to the manufacturer's instructions (Elabscience, catalog #E-BC-F070, E-BC-F069), with a final concentration of 1 µM. The plate was placed in a multimode microplate reader (Varioskan LUX, Thermo Scientific) for kinetic measurements. OCR was monitored using excitation and emission wavelengths of 405nm and 650 nm for 90 min at 2 min intervals, whereas ECAR was measured using excitation and emission wavelengths of 490 nm and 535nm for 120min at 4-min intervals. Fluorescence intensity was plotted against time, and OCR and ECAR were calculated from the linear portion of the curves between time points T1 and T2.

**Cell Counting Kit-8**. Cells were seeded at a density of 1000 cells per well in 96-well plates. At each time point, the medium was replaced 2h before measurement with 100µL of medium containing 10% CCK-8 reagent (Yeasen, catalog #40203ES76). After incubation for 2h, absorbance was measured at 450 nm. Measurements were taken at 24, 48, 72, 96, and 120 h. Optical density values were normalized to the 24 h time point to calculate relative proliferation rates.

**Transwell migration and invasion assays.** Polycarbonate membrane inserts with 8.0 µm pores (LABSELECT, catalog #14342) were required. For invasion assays, inserts were pre-coated with Matrigel (Corning, catalog #2334003) and hydrated prior to cell seeding. Cells were serum-starved for 24 h, resuspended in serum-free medium, and seeded into the upper chamber at a density of 2×10⁵ cells per insert, while the lower chamber was filled with complete medium containing serum. After incubation (16-24 h for 786O cells and 36-72 h for 769P cells), cells were fixed with 4% formaldehyde for 30min and stained with 0.1% crystal violet for 30min. Migrated or invaded cells were then visualized and imaged by fluorescence microscope.

**Subcutaneous implanted tumor model.** To assess the effect of cell proliferation *in vivo*, female immunodeficient NCG mice (NOD/ShiLtJGpt-Prkd^cem26cd52^ll2rg^em26cd22^/Gpt) at 4 weeks were purchased from GemPharmatech Co., Ltd. A total of 1×10⁷ LINC02532-overexpressing 786O cells or vector control cells were resuspended in 100 µL PBS and injected subcutaneously into the axillary region of each mouse. Tumors formed approximately one week after injection and exhibited measurable growth around four weeks, and tumor size was measured every three days using the formula: volume = length×width²/2. Mice were sacrificed when tumors in any group reached a volume of 1500 mm³ or when any tumor dimension (length, height, or width) was ≥ 15 mm, and subcutaneous tumors were harvested for further analysis.

**Vesicles isolation.** In brief, 5mL of whole blood was collected and processed within 2 h at 4 °C. Samples were first centrifuged at 1900 g for 10min at 4 °C, and approximately 1mL of the supernatant was collected and further centrifuged at 16000 g for 10 min at 4 °C to obtain serum. The serum was transferred to ultracentrifuge tubes and centrifuged at 100000 g for 120min at 4 °C. After discarding the supernatant, the vesicles were resuspended in pre-chilled PBS.

**Quantitative real-time polymerase chain reaction (RT-qPCR).** In brief, for RNA extraction, 200µL chloroform was added to the vesicles, followed by vortexing and incubation at room temperature. The mixture was centrifuged at 12000g and the upper aqueous phase was carefully collected. An equal volume of isopropanol was added, mixed thoroughly, and incubated at 4 °C for 1h to precipitate RNA. After centrifugation, 75% ethanol was added to wash RNA after discarding the supernatant. Genomic DNA was removed according to the kit's instructions (ABclonal, catalog #RK20429). Reverse transcription was performed at 42°C for 15min followed by 95 °C for 30s. Quantitative PCR was subsequently configured and fluorescence signals were acquired according to the kit's protocol (ABclonal, catalog #RK 21203).

## Results

### Patient cohorts and study design

**Figure 1:**
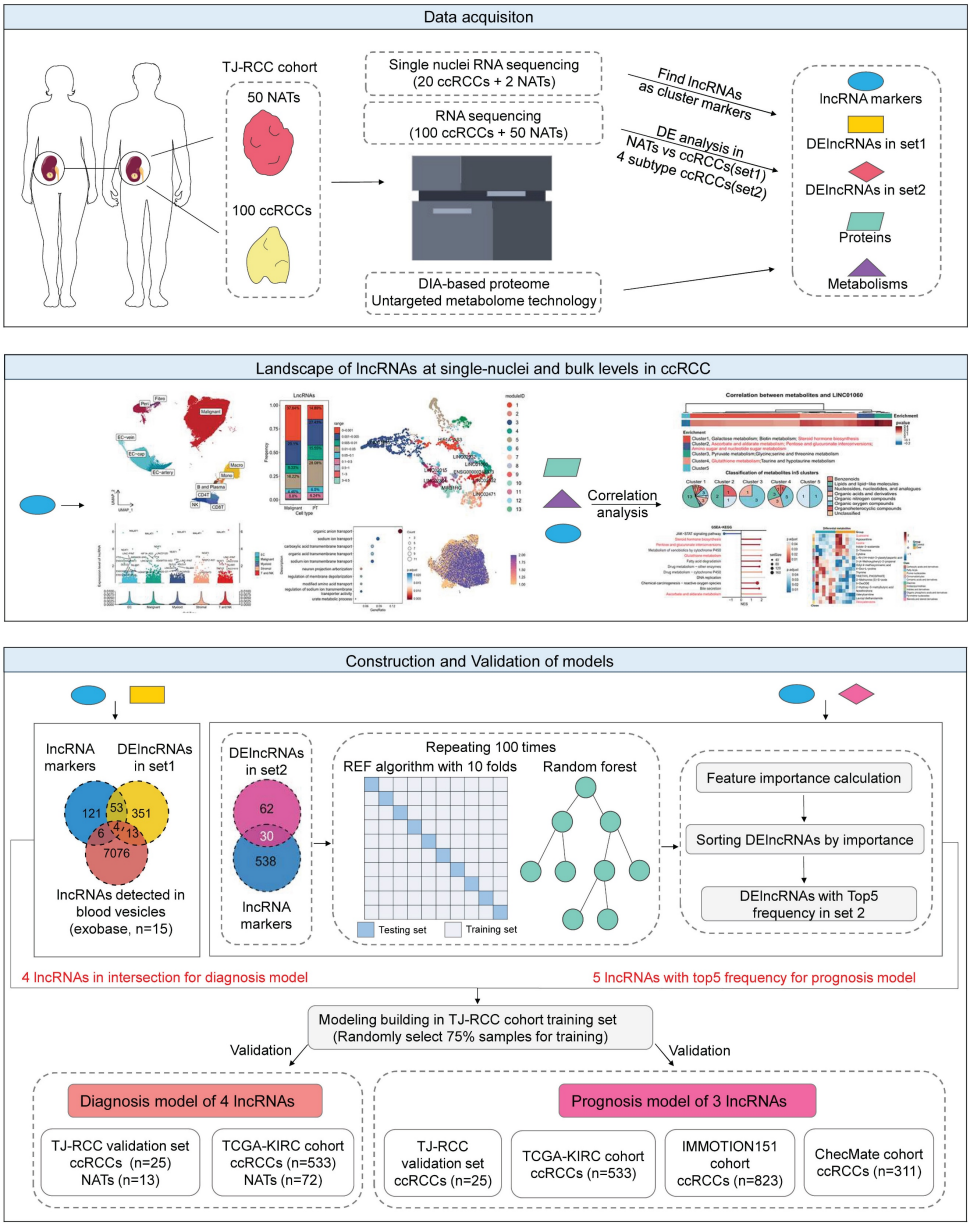
** Flow chart of the study and characteristics of lncRNAs in ccRCC.** Flowchart illustrating the procedural steps: 100 ccRCC samples and 50 NATs were collected. SnRNA-seq, bulk RNA-seq, proteomics, and untargeted metabolomics were integrated to identify the expression patterns and functional characteristics of lncRNAs. Diagnostic and prognostic models were then developed and validated across platforms. Abbreviations: NATs, adjacent normal tissues; snRNA-seq, single-nuclei RNA sequencing; bulk RNA-seq, RNA sequencing; lncRNAs, long noncoding RNAs.

The workflow was summarized in a flowchart (Figure 1). We collected 100 ccRCC samples and 50 NATs. We combined snRNA-seq, RNA sequencing (bulk-seq), proteomics, and untargeted metabolomics (both liquid chromatography and gas chromatography-mass spectrometry) to characterize lncRNA expression and function. The in-house cohort was centralized and randomly divided into independent training (n = 75) and testing (n = 25) sets.

For the diagnostic model, candidate variables were defined as the intersection of lncRNA markers derived from snRNA-seq, DElncRNAs between NATs and ccRCCs (set1), and blood-detected lncRNAs 41. For the prognostic model, variables were selected from lncRNA markers and DElncRNAs among the four prognostic ccRCC subtypes identified previously (set2) 12. During the validation phase, the diagnostic model was tested using the in-house cohort's validation set (25 ccRCC vs. 13 NATs) and the TCGA cohort (KIRC cohort, 533 ccRCC vs. 72 NATs; KIRP; KICH cohort). The prognostic model was validated in the in-house cohort's validation set (n = 25) and further assessed in the TCGA-KIRC cohort (n = 533), the IMMOTION151 cohort (n = 823) 15, and the CheckMate cohort (n = 311) 16.

### Characteristics of lncRNA expression in ccRCC

**Figure 2:**
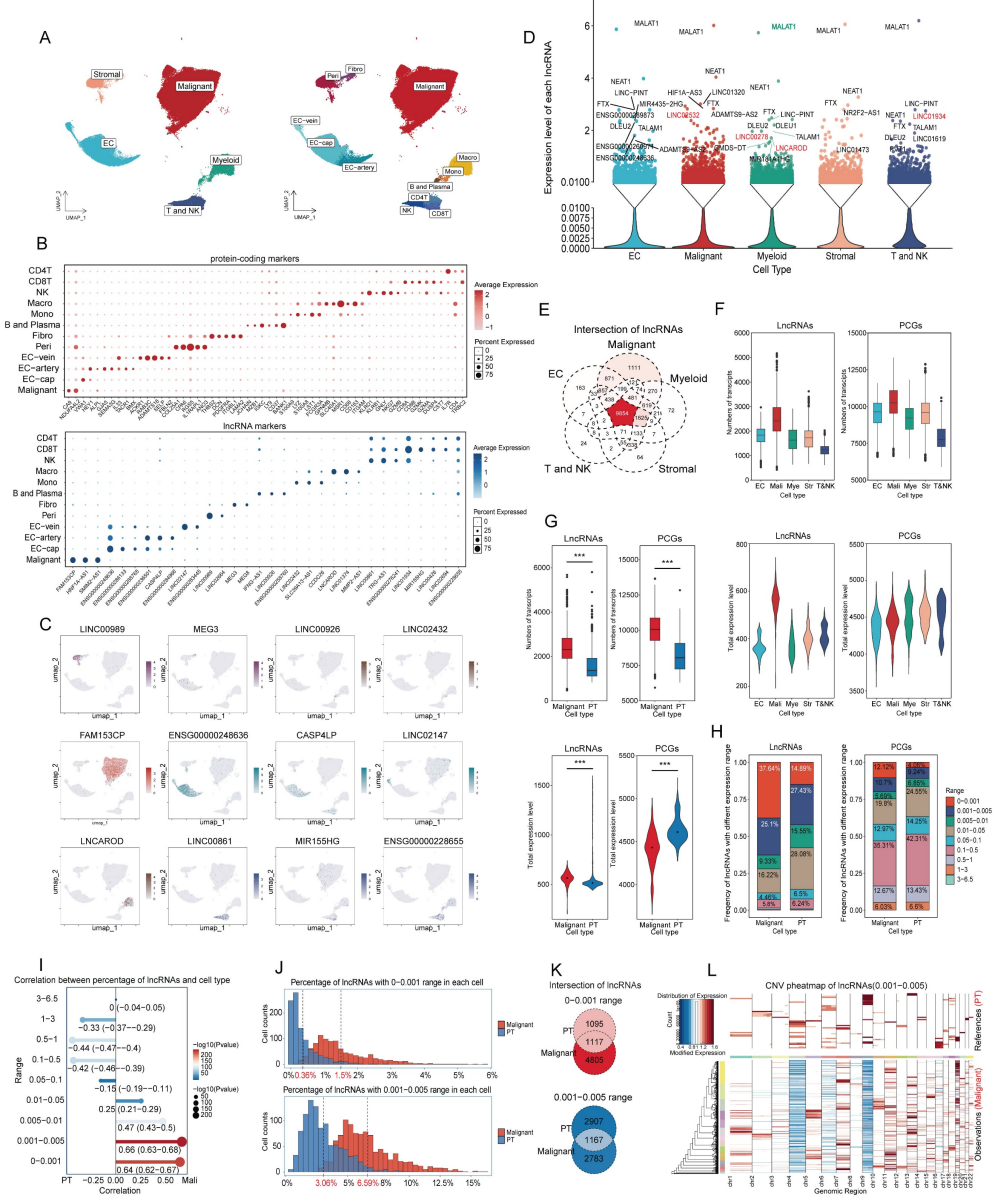
** Expression patterns of lncRNAs in ccRCC. (A)** UMAP plot of snRNA-seq data, colored by major cell types and subclusters.** (B)** Dot plot of protein-coding and representative lncRNAs with distinct expression patterns for each subcluster. **(C)** UMAP plot showing representative lncRNAs for each subcluster.** (D)** Expression levels of lncRNAs across the five major cell types. Expression levels were determined by dividing total expression by total metacell counts. LncRNAs with expression levels exceeding 0.01 were represented as dots, while those with levels greater than 1.5 were annotated. Notably, lncRNAs discussed below were highlighted in red. **(E)** Intersection of lncRNAs across five major cell types.** (F) and (G)** Bar and violin plots illustrate the number and total expression levels of lncRNAs across cell types, with these metrics calculated by summing values from all metacells, **(F)** across major cell types;** (G)** across malignant and PT nuclei.** (H)** Proportional composition of lncRNAs with different expression ranges in malignant and PT nuclei, calculated by dividing the number of lncRNAs within a specific range by the total number of lncRNAs. **(I)** Point biserial correlation analysis on the proportion of lncRNAs and nucleus types. Longer line segments indicate higher correlation, while larger dots indicate smaller P values. **(J)** Histogram showing the frequency distribution of low-expression lncRNAs in each metacell nucleus. **(K)** Intersection of low-expression lncRNAs in malignant and PT nuclei. **(L)** CNV heatmap of overlapping lncRNAs with expression levels between 0.001 and 0.005 in malignant nuclei, using PT nuclei as the reference. Abbreviations: UMAP, uniform manifold approximation and projection; lncRNAs, long noncoding RNAs; CNV, copy number variations.

SnRNA-seq was performed on 20 ccRCC samples and 2 NATs. 94703 single nuclei were classified into 5 major cell types and 12 subclusters after quality control (Figure 2A). Classic protein-coding markers and representative lncRNAs with distinct expression patterns were shown in Figure 2B-C, demonstrating satisfactory performance in subcluster identification of lncRNAs. To quantitatively evaluate the clustering performance, nuclei were normalized and reduced dimensionally using either classic markers or selected lncRNA as features. Notably, compared to classic markers, lncRNA achieved higher silhouette coefficient (0.1006 vs. 0.0359), Calinski-Harabasz index (1131.01 vs. 236.51), and lower Davies-Bouldin index (1.8090 vs. 3.3015), which demonstrated that lncRNAs provided reliable resolution for subcluster identification (Figure S2A).

Because of matrix sparsity and low lncRNA abundance, nuclei with similar profiles were merged into one unit called metacells for downstream analysis 25. Across the five major cell types (Figure 2D), most lncRNAs showed extremely low expression. LncRNAs with expression levels exceeding 0.01 were displayed as dots, and those with levels above 1.5 were annotated. Several lncRNAs were broadly expressed (MALAT1, NEAT1, FTX, and LINC-PINT), suggesting roles in essential cellular processes, whereas others showed cell type-specific patterns. Additionally, malignant nuclei contained the largest set of unique lncRNAs (Figure 2E) and displayed the highest overall transcriptional activity, measured by both lncRNA count and expression level compared to other nuclei (Figure 2F).

Given that ccRCC arises from tubular epithelial cells 52, we next compared malignant nuclei with PT nuclei from NATs. After annotation, quality control, dimension reduction, and clustering, malignant nuclei exhibited significantly higher numbers and expression counts of lncRNAs (Figure 2G). Notably, the number of lncRNAs in malignant nuclei was nearly twice that in PT nuclei, whereas overall expression levels increased only modestly, reflecting widespread but low-level transcription. To validate this finding, lncRNAs were stratified into nine ranges according to average expression per metacell. Average expression was calculated as total expression divided by the number of nuclei with nonzero counts (Table S3). Proportional analysis showed that low-abundance lncRNAs (0-0.001 and 0.001-0.005) were more prevalent in malignant nuclei than protein coding genes (PCGs) and accounted for nearly 62.7%, whereas they comprised 42.32% in PT nuclei. The 0-0.005 range was particularly enriched in malignant nuclei (37.64%) compared with PT nuclei (14.89%). (Figure 2H). Point biserial correlation analysis demonstrated a positive association between low-abundance lncRNAs and malignant nuclei (Figure 2I). To further characterize the expression patterns of low-abundance lncRNAs, we plotted frequency distributions of low-abundance lncRNAs per metacell (Figure 2J). The average frequency was lower in PT nuclei (0.36% for 0-0.001, 3.06% for 0.001-0.005) and higher in malignant nuclei (1.5% for 0-0.001, 6.59% for 0.001-0.005). The distribution peak was concentrated and steep in PT nuclei but diffuse and gradual in malignant nuclei. Besides, Intersection analysis revealed minimal overlap between low-abundance lncRNAs in PT and malignant nuclei (Figure 2K). This suggested that PT nuclei tended to transcribe fewer lncRNAs at higher levels, whereas malignant nuclei express a broader set at lower levels.

To further explore this phenomenon, we analyzed copy number variations (CNVs) using overlapping low-abundance lncRNAs (Figure 2L, S1B-S1I). The heatmap revealed that CNVs were concentrated in some PT nuclei but dispersed across most malignant nuclei, potentially explaining the broader transcription of low-abundance lncRNAs in malignancy. Additionally, the CNV scores for low-abundance lncRNAs were lower in malignant nuclei than in PT nuclei, whereas scores for high-expression lncRNAs (> 0.05) were slightly higher (Figure S1B-S1I). Considering that CNV scores were calculated from absolute values per metacell, it suggested that PT nuclei tended to transcribe lncRNAs at higher levels. These results suggested that malignant nuclei transcribe more low-abundance lncRNAs, while PT nuclei favor fewer but highly expressed lncRNAs with individual variability. Overall, the low-abundance lncRNAs may reflect the dispersed and widespread CNV landscape characteristic of malignancy.

### Pipeline on inference of lncRNA function in ccRCC

Because of the non-coding nature, functional inference of lncRNAs could be challenging. In this study, we applied hdWGCNA to each major cell type to identify co-expressed PCGs and lncRNAs, where co-expression genes were assigned to the same module 25. Focusing on highly expressed lncRNAs, their associated biological processes were inferred via GO enrichment of proteins in the same module. Further, bulk transcriptome data were integrated with metabolomic and proteomic data to infer potential lncRNA-mediated pathways and molecular interactions (Table S5). Using this pipeline, we preliminarily identified five lncRNAs that may drive metabolic and immunological programs in ccRCC, underscoring the biological relevance of this integrative approach.

### Malignant nuclei

**Figure 3:**
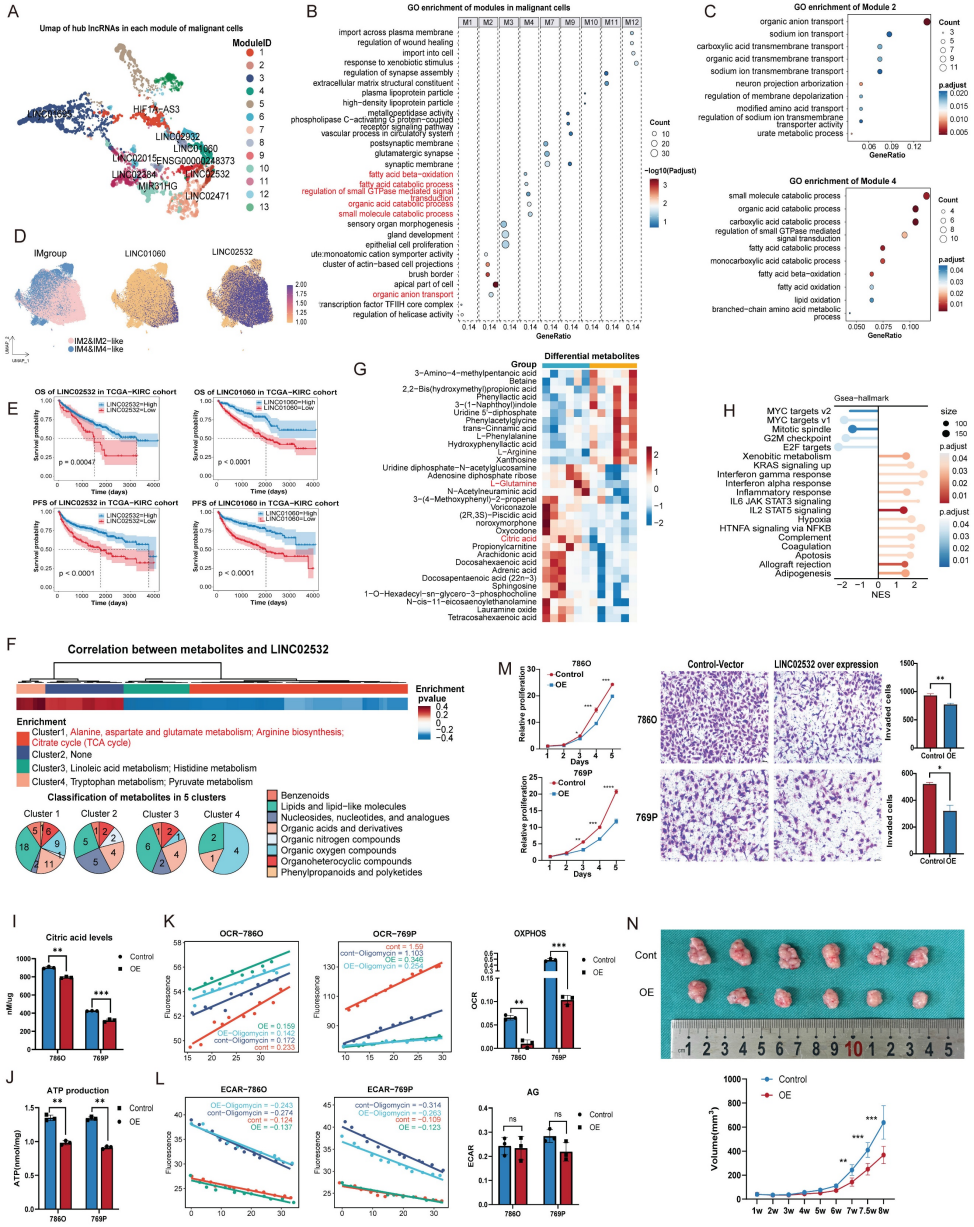
** Function characteristics of lncRNAs in malignant nuclei. (A)** UMAP plot of hub lncRNAs in malignant nuclei colored by module. LncRNAs with high kME values were identified as the hub genes of a module. **(B)** GO enrichment analysis of PCGs in each malignant nucleus module. **(C)** GO enrichment analysis of biological processes was conducted for PCGs in modules 2 and 4, which were associated with metabolism. **(D)** Expression of LINC01060 and LINC02532 in harmony-adjusted UMAP of malignant nuclei in snRNA-seq, colored by IM2&IM2-like and IM4&IM4-like identities. **(E)** OS and PFS of LINC02532 and LINC01060 in the TCGA-KIRC cohort.** (F)** Heatmap of lncRNA-metabolite pairs with a correlation greater than 0.3, annotated with the enrichment and super class distribution of metabolites corelated with LINC02532. **(G)** Heatmap of differential metabolites between the LINC02532-overexpressing and wild-type 786O cell line. Metabolites consistent with the pipeline's inferred conclusions were highlighted in red.** (H)** GSEA analysis of DEGs between the LINC02532-overexpressing 786O cell line and the wild-type cell line.** (I) and (J)** Intracellular citric acid levels **(I)** and ATP **(J)** across control and overexpressing cells. **(K)** OCR and OXPHOS capacity in control and overexpressing cells. The slope of fluorescence over time reflected OCR, and the difference between the oligomycin-treated and untreated groups indicated OXPHOS capacity. **(L)** ECAR and AG in control and overexpressing cells. The slope of fluorescence over time reflected ECAR, and the oligomycin-treated group indicated ECAR levels. **(M)** Cell proliferation detected by CCK8 and Transwell assays demonstrated reduced cell viability and invasiveness. **(N)** Subcutaneous xenograft tumor model established using LINC02532-overexpressing and control 786O cells in female immunodeficient NCG mice for 8 weeks (n = 6). Abbreviations: UMAP, uniform manifold approximation and projection; lncRNAs, long noncoding RNAs; GO, Gene Ontology; PCGs, protein-coding genes; OS, overall survival; PFS, progression-free survival; GSEA; gene set enrichment analysis; DEGs, differential expression genes; OCR, Oxygen consumption rate; OXPHOS, oxidative phosphorylation; ECAR, extracellular acidification rate; AG, Anaerobic Glycolysis.

The hub lncRNAs in each module were identified by kME values and visualized on UMAP (Figure 3A), with all genes and corresponding kME values listed in Table S4. LINC02532 represented module 2, enriched in metabolic processes and mainly linked to anion transport, whereas LINC01060 corresponded to module 4, associated with catabolism and oxidation (Figures 3B-C). Consistent with our previous study 12, IM2 and IM2-like ccRCCs characterized by high metabolic activity and favorable prognosis, showed elevated expression of both two lncRNAs compared with IM4 and IM4-like tumors (Figure 3D). High expression of LINC02532 and LINC01060 correlated with improved survival in the TCGA-KIRC cohort (Figures 3E, S2A-B).

Metabolites associated with LINC02532 and LINC01060 were identified through correlation analyses of transcriptome and metabolome data (Table S5). Most LINC02532-negatively associated metabolites were enriched in glutamate and arginine biosynthesis, as well as the citrate cycle (also called tricarboxylic acid cycle, TCA cycle) (Figure 3F). To validate these findings, we generated a LINC02532 over-expression 786O cell line for metabolomic profiling, which shown upregulation of L-Arginine, downregulation of L-Glutamine and citric acid (Figure 3G), and GSEA of transcriptome revealed enrichment of stress response pathways, cell cycle arrest, and apoptosis (Figure 3H).

Measurement of citric acid levels confirmed a reduction in LINC02532 over-expression cells (Figure 3I). Considering that TCA cycle was essential for oxidative phosphorylation (OXPHOS) and adenosine triphosphate (ATP) production, we measured intracellular ATP levels in LINC02532 over-expression cells and observed a significant reduction in ATP (Figure 3J). To further investigate the source of ATP reduction, we assessed the OCR (representing OXPHOS) and the ECAR (representing glycolysis) (Figure 3K-L). The marked decrease in OCR with an unchanged ECAR indicated that ATP reduction primarily resulted from impaired OXPHOS rather than anaerobic glycolysis. In addition, CCK-8 and Transwell experiments demonstrated significantly reduced proliferative and invasive capacities of LINC02532 over-expression cells (Figure 3M). To evaluate the effect of over-expression LINC02532 *in vivo*, a subcutaneous xenograft model was established in immunodeficient NCG mice. Tumors derived from LINC02532 over-expression cells exhibited significantly smaller volumes compared with controls, indicating suppressed tumor proliferation *in vivo* (Figure 3N). Collectively, these results suggested that LINC02532 over-expression induced metabolic reprogramming and cellular stress.

**Figure 4:**
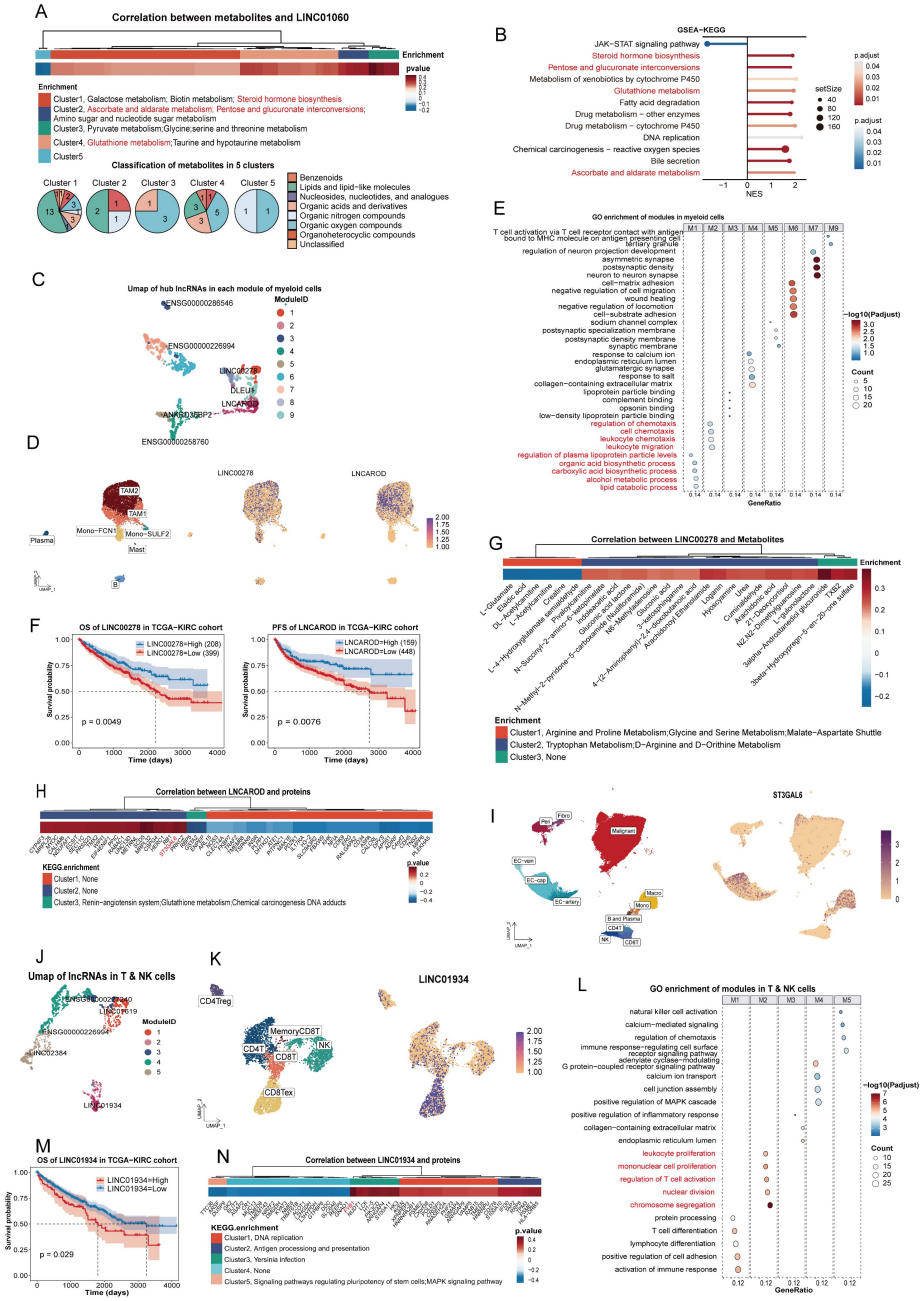
** Function characteristics of lncRNAs in immune nuclei. (A)** Heatmap of lncRNA-metabolite pairs with a correlation greater than 0.3, annotated with the enrichment and super class distribution of metabolites corelated with LINC01060. **(B)** GSEA analysis of DEGs between the LINC01060-overexpressing 769P cell line and the wild-type cell line. Metabolic pathways consistent with the pipeline's inferred conclusions are highlighted in red. **(C)** UMAP plot of hub lncRNAs in myeloid nuclei, colored by module. **(D)** Expression of LINC00278 and LNCAROD in the harmony-adjusted UMAP of myeloid nuclei in snRNA-seq, colored by subcluster. **(E)** GO enrichment analysis of PCGs in each myeloid nucleus module.** (F)** OS of LINC00278 and PFS of LNCAROD in the TCGA-KIRC cohort. **(G)** Heatmap of LINC00278-metabolite pairs with correlations greater than 0.2, annotated with metabolite enrichment. **(H)** Heatmap of LNCAROD-protein pairs with correlations greater than 0.3, annotated with KEGG enrichment of proteins; ST3GAL6 is highlighted in red. **(I)** UMAP plot showing the distribution of ST3GAL6 across 12 subclusters.** (J)** UMAP plot of hub lncRNAs in T and NK nuclei, colored by module. **(K)** Expression of LINC01934 in the harmony-adjusted UMAP of T and NK nuclei in snRNA-seq, colored by subcluster. **(L)** GO enrichment analysis of PCGs in each T and NK nucleus module. **(M)** OS of LINC01934 in the TCGA-KIRC cohort. **(N)** Heatmap of LINC01934-protein pairs with correlations greater than 0.3, annotated with KEGG enrichment of proteins; FYB1 is highlighted in red. Abbreviations: UMAP, uniform manifold approximation and projection; lncRNAs, long noncoding RNAs; OS, overall survival; PFS, progression-free survival; GO, Gene Ontology; KEGG, Kyoto Encyclopedia of Genes and Genomes.

Similarly, LINC01060 influenced multiple metabolic pathways, including steroid hormone biosynthesis and pentose/glucuronate interconversions, which were validated by GSEA in 769P cells (Figure 4A-B). According to previous study, LINC01060 may contribute to metabolic processes through the MZF1/c-Myc/HIF1α axis 53.

### Myeloid nuclei

The UMAP of the myeloid module was shown in Figure 4C. As a hub lncRNA in module 1, LINC00278 was broadly expressed in myeloid nuclei (Figure 4D) and associated with acid and lipid metabolism processes (Figure 4E), suggesting a role in core metabolic functions of myeloid cells. Survival analysis indicated that low expression of LINC00278 correlated with poorer prognosis (Figure 4F, Figure S1Ja). Integration of transcriptomic and metabolomic data further indicated that LINC00278 was primarily linked to amino acid metabolism (Figure 4G). Previous studies have shown that LINC00278 encodes the micropeptide YY1BM 54, which interacts with YY1 to regulate key metabolic enzymes 55, suggesting a potential mechanism for this regulation.

LNCAROD, identified as a macrophage marker (Figure 2B, 4D), may contribute to macrophage chemotaxis (Figure 4E). The progression-free survival (PFS) differed significantly between high and low-expression groups, but overall survival (OS) did not (Figure 4F, S1Jb), suggesting that LNCAROD may influence early treatment response. Integrating proteomic data revealed a positive correlation between LNCAROD and ST3GAL6 (cor = 0.36) (Figure 4H, Table S5). As a member of the sialyltransferase family, ST3GAL6 was crucial for the sialylation of selectin ligands 56. Furthermore, snRNA-seq data showed that ST3GAL6 was expressed in both macrophage and endothelial cell nuclei, indicating that LNCAROD may regulate macrophage migration in ccRCC by modulating ST3GAL6 (Figure 4I).

### T and NK nuclei

Only 5 modules were identified In T and NK nuclei (Figure 4J). As shown in Figure 2K, LINC01934 was particularly enriched in exhausted CD8+ nuclei, suggesting a potential role in regulating T cell activation (Figure 4L). Additionally, survival analysis indicated that high LINC01934 expression may be detrimental to patients (Figure 4M, S1Jc). Additionally, integration of proteomic data revealed that FYB1 exhibited the strongest correlation with LINC01934 (cor = 0.50) (Figure 4N, Table S5). FYB1, a key regulator of T cell receptor signaling that has been extensively studied 57, may provide mechanistic insight into the function of LINC01934 in exhausted CD8+ cells.

### Construction and validation of the diagnosis model of ccRCC

**Figure 5:**
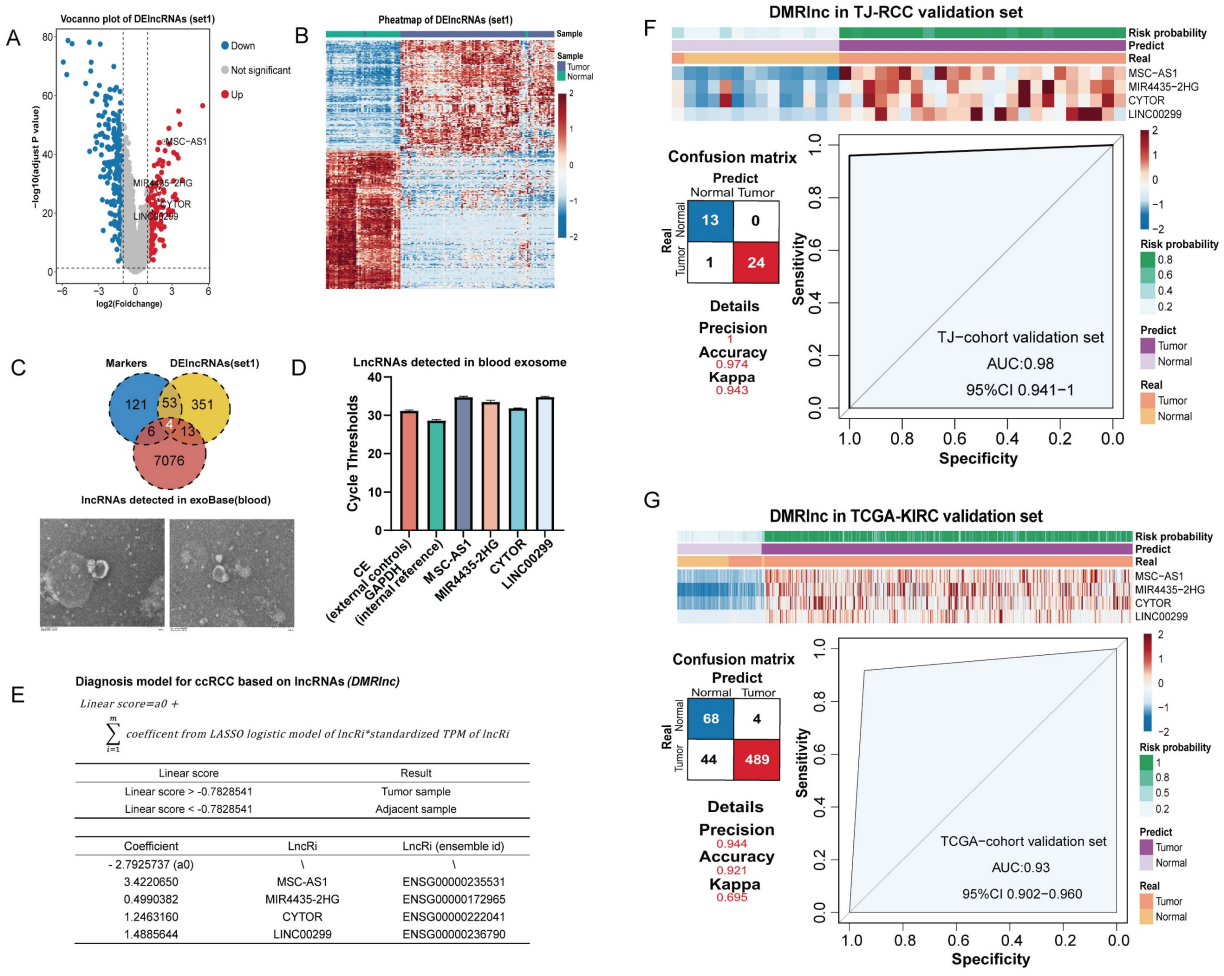
** Construction and validation of DMRlnc in ccRCC. (A)** Volcano plot showing the log2(fold change) of DElncRNAs (set1) with adjusted p-values < 0.05, highlighting DMRlnc features.** (B)** Heatmap of unsupervised hierarchical clustering of DElncRNAs (set1). **(C)** Intersection of lncRNA markers, DElncRNAs (set 1), and lncRNAs detected in blood from the exoRBase dataset, used to define DMRlnc features. Representative transmission electron microscopy images of extracellular vesicles isolated from ccRCC patients were shown. **(D)** RT-qPCR validation of four selected DMRlnc features using an exogenous nematode RNA as an external reference and GAPDH as an internal control. **(E)** Parameters and features of the DMRlnc model.** (F)** Heatmap, confusion matrix, and ROC curve illustrating the expression patterns and predictive performance of DMRlnc in the TJ-RCC validation cohort.** (G)** Heatmap, confusion matrix, and ROC curve illustrating the expression patterns and predictive performance of DMRlnc in the TCGA-KIRC validation cohort. Abbreviations: DMRlnc, diagnosis model for ccRCC based on lncRNAs; DElncRNAs (set1), differentially expressed lncRNAs between ccRCC and normal adjacent tissues; RT-qPCR, quantitative real-time polymerase chain reaction; ROC, receiver operating characteristic.

To seek a less invasive diagnostic approach, we aimed to construct a model based on lncRNA. The TJ-RCC cohort were randomly divided into independent training (n = 75) and testing (n = 25) sets for model building. 351 DElncRNAs were identified to differentiate ccRCC from NATs (187 upregulated and 164 downregulated) (Figure 5A). The heatmap demonstrated that DElncRNAs (set1) effectively distinguished ccRCC from NATs (Figure 5B). To enhance clinical generalizability and minimize feature complexity, we incorporated exoRBase, an external database containing RNA-seq profiles of blood extracellular vesicles from 15 KIRC samples for feature screening 41. Four features (MSC-AS1, MIR4435-2HG, CYTOR, LINC00299) were selected from the intersection of lncRNA markers, DElncRNAs (set1), and lncRNAs detected in blood (Figure 5C). To validate the presence of these lncRNAs in circulation, extracellular vesicles were isolated from the peripheral blood of ccRCC patients, and the expression levels of the four lncRNAs were quantified using external and internal controls by RT-qPCR (Figure 5C-D). Based on the actual disease status of the in-house cohorts training set, a logistic regression model was developed using these four features to identify ccRCC, which was termed DMRlnc (Figure 5E). None of the features were eliminated during model fitting, indicating stable coefficients and minimal risk of overfitting. The optimal threshold for distinguishing tumor from normal samples was determined by maximizing Youden's J statistic 42.

DMRlnc was validated in the TJ-cohort's testing set (Figure 5F), achieving an AUC of 0.98 and a Kappa coefficient of 0.943, indicative of near-perfect agreement. In the TCGA-KIRC cohort, DMRlnc further demonstrated robust performance, with a precision of 0.944, an accuracy of 0.921, and an AUC of 0.93 (Figure 5G). Although DMRlnc was developed based on ccRCC, we sought to evaluate its applicability in other kidney cancer subtypes. In kidney renal papillary cell carcinoma (pRCC, TCGA-KIRP), DMRlnc achieved a precision of 0.656, an accuracy of 0.87, and an AUC of 0.77, which indicating acceptable performance (Figure S2A). In contrast, its performance in chromophobe renal cell carcinoma (chRCC, TCGA-KICH) was modest (Figure S2B), which may be due to differences in the cellular origin of these cancers 52,58-60, suggesting that DMRlnc was more suitable for ccRCC.

### Construction and validation of the prognosis model of ccRCC

**Figure 6:**
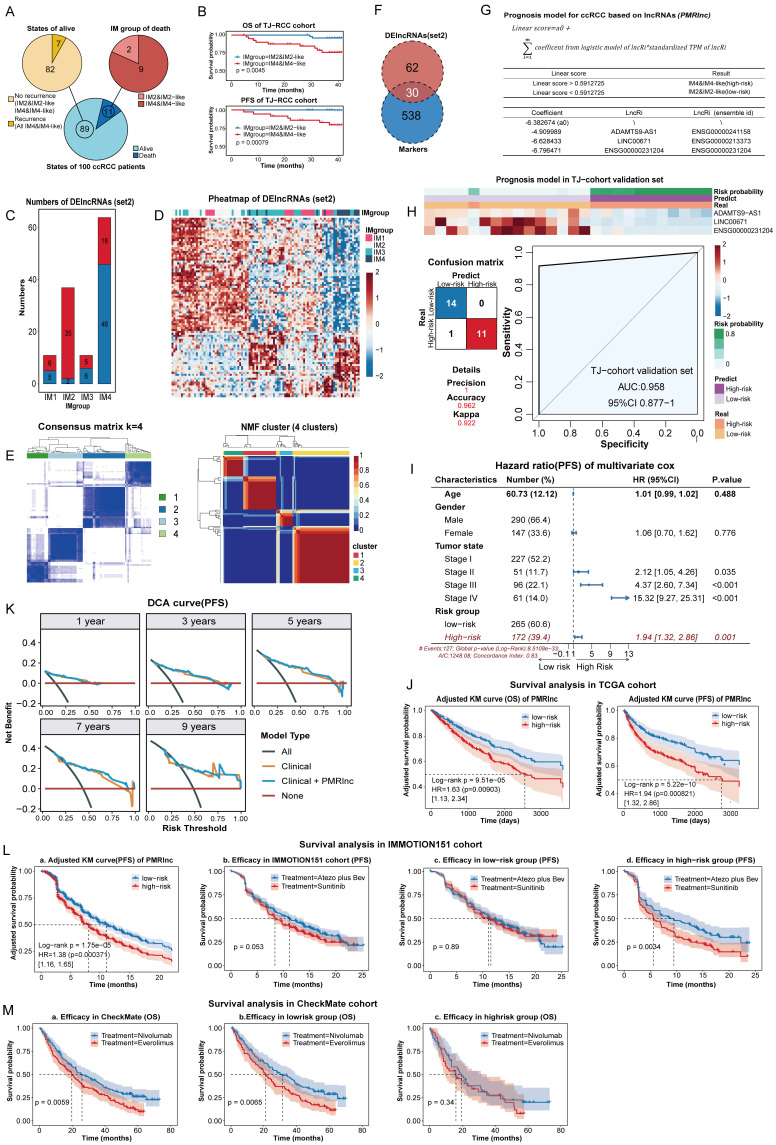
** Construction and validation of PMRlnc in ccRCC. (A)** Three-year follow-up results of the TJ-RCC cohort. **(B)** Survival analysis on the three-year follow-up of the TJ-RCC cohort.** (C)** Bar plot illustrating the number of DElncRNAs (set2) across four subtypes. **(D)** Heatmap of unsupervised hierarchical clustering of DElncRNAs (set2) in ccRCC tissues. **(E)** Consensus matrix and NMF clusters based on lncRNA markers, indicating an optimal k=4.** (F)** Intersection of lncRNA markers and DElncRNAs (set2) used to select features for PMRlnc.** (G)** Parameters and features of the PMRlnc model. **(H)** Heatmap, confusion matrix, and ROC curve showing the expression patterns and consistency of PMRlnc in the TJ-RCC validation cohort. **(I)** Forest plot of HR for PFS derived from multivariate cox regression including PMRlnc and clinical variables in the TCGA-KIRC cohort. **(J)** OS and PFS of two risk groups distinguished by PMRlnc in the TCGA-KIRC cohort.** (K)** DCA comparing the clinical model alone with the combined clinical + PMRlnc model for PFS prediction. **(L) a:** PFS of high-risk and low-risk groups identified by PMRlnc in the IMmotion151 cohort; **b-d:** PFS comparison between treatment groups (atezo plus bev vs. sunitinib) in the entire IMmotion151 cohort (b), the low-risk group (c), and the high-risk group (d). **(M) a-c:** OS comparison between treatment groups (everolimus vs. nivolumab) in the CheckMate cohort (a), the low-risk group (b), and the high-risk group (c). Abbreviations: PMRlnc, prognosis model for ccRCC based on lncRNAs; DElncRNAs (set2), differentially expressed lncRNAs among four ccRCC subtypes; NMF, non-negative matrix factorization; ROC, receiver operating characteristic; HR, hazard ratio; DCA; decision curve analysis; OS, overall survival; PFS: progression-free survival; atezo plus bev; atezolizumab plus bevacizumab.

In our previous study, ccRCCs were classified into four immune subtypes, with IM1/IM3 tissues further subdivided into IM2-like or IM4-like 12. In the three years follow-up, 9/11 fatal and all 7 recurrent cases occurred in IM4/IM4-like (Figure 6A), while survival analysis confirmed their poorer outcomes (Figure 6B). Thus, IM4/IM4-like were defined as high-risk, while IM2/IM2-like represented the low-risk group. We therefore constructed a prognostic model based on in-house survival data to support clinical decision-making.

To identify specific features, DElncRNAs (set2) were determined for each subtype versus the other three. The largest numbers of DElncRNAs detected in IM2 and IM4 indicated marked differences (Figure 6C). A heatmap confirmed that DElncRNAs(set2) clearly distinguished IM2 and IM4, with IM2/IM3 clustering in between (Figure 6D), demonstrating clear separation between IM2/IM2-like and IM4/IM4-like. In addition, lncRNAs identified across 12 subtypes classified 100 samples into four clusters with optimal performance using consensus 35 and NMF clustering 36 (Figure 6E, S2C-D). Therefore, candidate variables were refined by intersecting with DElncRNAs (set2) and lncRNA markers (Figure 6F). To remove less informative features and reduce overfitting, RFE with 10-fold cross validation was repeated 100 times on the intersection. Five features with the highest selection frequency (EMOX2OS, ADAMTS9-AS1, LINC00671, DRAIC, and ENSG00000231204) were subsequently used to construct the PMRlnc model using logistic regression, and three (ADAMTS9-AS1, LINC00671, ENSG00000231204) were remained on model significance (Figure 6G). The PMRlnc model demonstrated strong consistency in the TJ-cohort validation set, yielding a Kappa coefficient of 0.922 and an AUC of 0.958 (Figure 6H).

In the TCGA cohort, multivariate cox analysis shown that risk group stratified by PMRlnc was an independent predictor of progression with hazard ratio (HR) of 1.94 (Figure 6I, S3A). After adjusting for confounding factors, the KM survival curves shown that low-risk patients exhibited significantly longer OS and PFS (Figure 6J). We further evaluated whether integrating PMRlnc with conventional clinical variables could improve prognostic accuracy. Age, gender, and tumor stage were added to the clinical models, and the combined clinical + PMRlnc model was assessed in the TCGA cohort. For discrimination, the time-dependent ROC curves, net reclassification improvement (NRI), and integrated discrimination improvement (IDI) at each year indicated enhanced ability to stratify patients by risk (Figure S3B). For calibration and model fit, the closer calibration curves and small time-dependent barrier scores shown that combining PMRlnc improved prediction accuracy (Figure S3C). Decision curve analysis further suggested that adding PMRlnc increased the clinical utility of the models, particularly in the long term (Figure 6K, S3D). Overall, these results indicated that PMRlnc enhanced the prognostic value of conventional clinical features. In addition, the applicability of PMRlnc was explored in other kidney cancers. Similar to DMRlnc, PMRlnc shown predictive value in TCGA-KIRP but had limited utility in TCGA-KICH. PMRlnc remained an independent risk factor (HR of 2.82 in OS, HR of 1.28 in PFS) and effectively stratified patients into distinct risk groups (Figure S3E).

To further assess the prognostic performance of PMRlnc, we applied it to the IMMOTION151 cohort 15, a phase 3 trial comparing atezolizumab plus bevacizumab (atezo plus bev) with sunitinib in 823 treatment-naive RCC patients. In the IMMOTION151 cohort, which included only PFS data, PMRlnc served as an independent predictor, and patients in the low-risk group had longer PFS compared to the high-risk group (Figure 6La, S4A). Although overall efficacy of atezo plus bev was nearly equivalent to that of sunitinib (Figure 6Jb), high-risk patients identified by PMRlnc receiving atezo plus bev showed improved PFS compared to those on sunitinib, whereas no difference was observed in the low-risk group (Figure 6Jc-Jd). Additionally, PMRlnc was further evaluated in the CheckMate cohort, comprising 311 RCC patients previously received anti-angiogenic regimens 16. In this cohort, PMRlnc was not an independent predictor and PFS did not differ significantly across any subgroups, possibly due to prior anti-angiogenic and immune treatment (Figure S4B,4C). OS also did not differ significantly between risk groups (Figure S4D). Notably, the nivolumab-treated patients demonstrated longer OS than those treated with everolimus (Figure 6Ma). Further subgroup analysis indicated that nivolumab was associated with superior OS in the low-risk group not high-risk group (Figure 6Mb,6Mc).

In summary, PMRlnc effectively stratified RCC patients into risk groups, with patients in the low-risk group may derive greater benefit from nivolumab, whereas those in the high-risk group achieve improved outcomes with atezolizumab plus bevacizumab. Consequently, the PMRlnc may inform personalized therapy decisions and improve treatment efficacy and patient outcomes.

## Discussion

LncRNAs have been implicated in tumor proliferation 61, metastasis 62 and drug resistance 63 in kidney cancer. In this study, we integrated snRNA-seq, bulk RNA-seq, proteomics, and untargeted metabolomics to provide a comprehensive characterization of lncRNA expression and demonstrate their potential as both biological regulators and clinical tools. Malignant cells exhibited a broader but lower abundance of lncRNAs, likely reflecting widespread copy number alterations, whereas low-abundance lncRNAs in normal epithelial cells showed stronger individual specificity. We also provided a pipeline to preliminary infer the function of lncRNAs with high expression levels and identified five lncRNAs with potential significance linked to metabolism and immunity. Additionally, we established two clinically applicable models to improve early detection, risk stratification, and therapeutic decision-making in ccRCC, and achieved robust performance across multiple cohorts.

In our study, malignant cells exhibited broader but low-abundance lncRNA expression, whereas low-abundance lncRNAs in normal epithelial cells shown more individual variability. Since somatic mutations occurred in both contexts but were more frequent in cancer, this may explain the broader presence of such transcripts in tumors 64. Regarding the function of lncRNAs, their regulatory capacity did not scale linearly with expression and may require only a minimal expression threshold. For instance, as few as nearly 100 molecules of Xist were sufficient to silence an entire X chromosome 65. Consistently, Xist was detected at levels of 0.005-0.01 in our dataset (Table S3) and was retained within hdWGCNA co-expression modules (Table S4). However, only a small fraction of low-abundance lncRNAs (179 of 9872) could be assigned to hdWGCNA co-expression modules in our study (Table S4), whereas the majority were excluded due to high expression noise, severe dropout in single-cell data, and non-linear expression patterns 25. Therefore, low-abundance lncRNAs not captured by co-expression modules were more likely to represent transcriptional byproducts of chromosomal instability and variation with limited biological significance rather than coordinated regulatory programs 66. Nevertheless, their potential biological functions required additional evidence, including structural, spatial localization and experimental validation.

Given the low expression of circulating tumor cells 67, the short half-life of cfDNA (16min-2.5h) 68, individual variability in metabolites 69, and cost considerations for protein detection 70, circulating cell-free RNA (cfRNA) has emerged as a favorable class of biomarkers 71. Notably, lncRNAs encapsulated in extracellular vesicles from blood or urine offer enhanced specificity and stability, making them favorable candidates for tumor diagnosis and prognosis, especially with the rapid advances in liquid biopsy technologies 11,72,73. In this study, we focused on lncRNAs with relatively higher expression levels that were consistently detectable across patients' blood to build clinical models. Because low-abundance lncRNAs in the circulation remained challenges to measure with sufficient sensitivity and reproducibility 74, and may be substantially diluted by abundant non-tumor-derived RNA or degraded by circulating RNases 73, leading to unstable and variable measurements under current technical limitations in cfRNA detection. Moreover, the fluctuations in expression levels of low-abundance lncRNAs made the results highly susceptible to minor variations in sample handling and RNA extraction procedures 75. Consequently, low-abundance circulating lncRNAs were less suitable as biomarkers especially in liquid biopsy under current technical constraints.

We aimed to develop a diagnostic model applicable before biopsy or surgery. To ensure clinical feasibility and stability, DMRlnc features were screened using an external blood vesicle RNA-seq dataset, only lncRNAs detected in all 15 samples were selected to ensure robust detectability 41,73. All four features are upregulated in ccRCC, where MSC-AS1 76, MIR4435-2HG 77, and CYTOR 78 widely recognized as cancer biomarkers. While most published diagnostic models target general RCC rather than ccRCC-specific features, DMRlnc integrates multiple lncRNAs, achieving higher predictive accuracy than any single gene 79-82. Importantly, DMRlnc has been developed in a large in-house cohort and validated in external datasets, highlighting its robustness and ccRCC specific clinical utility. Previous prognostic models for ccRCC, such as Wang *et al.*'s 17-lncRNA Cox regression model 83 and Leibovich *et al.*'s subtype-specific clinicopathologic models 84, were either complex or lack of validation in external datasets cohort and reliant on post-surgical clinicopathologic data. In contrast, PMRlnc was concise and practical, requiring only three features from a small surgery tissue sample. Importantly, PMRlnc functioned as an independent prognostic factor in RCC, retaining predictive value after adjustment for conventional clinical variables, including age, gender, tumor stage, and grade in our study. Moreover, integrating PMRlnc into existing clinical factors improved risk stratification, reflected by enhanced discrimination (time-dependent ROC, NRI, IDI), improved calibration, and increased clinical utility in DCA. These findings suggested that PMRlnc could complement traditional prognostic indicators, providing finer resolution for identifying patients at higher or lower risk of progression.

To explore the applicability of DMRlnc and PMRlnc across different kidney cancers, we evaluated their performance in TCGA-KIRP and TCGA-KICH cohorts. However, both DMRlnc and PMRlnc showed reasonable predictive value in KIRP but were less effective in KICH. Given that DMRlnc and PMRlnc were developed based on lncRNAs with characteristics of cell specific expression, differences in the cellular origins of kidney cancers may explain the observed variation in performance. ccRCC originated from proximal tubule cells 52, while KIRP may arise from the proximal tubule cells or from principal cells of the collecting ducts 60. In contrast, KICH was more likely derived from distal convoluted tubule cells, which were less similar to those giving rise to ccRCC or pRCC 58,59.

Besides, validation in the IMMOTION151 and CheckMate cohorts showed that high-risk RCC patients benefit more from atezolizumab plus bevacizumab, whereas low-risk patients respond better to nivolumab. According to our previous study 12, high-risk tumors (IM4/IM4-like) were enriched in angiogenesis-related pathways, whereas low-risk tumors (IM2/IM2-like) exhibited enhanced metabolic activity and T-cell infiltration. This may explain why the combination of the VEGFR inhibitor (bevacizumab) improved PFS in the high-risk group (IM4/IM4-like), while the PD-L1 inhibitor (nivolumab) was more effective in the low-risk group (IM2/IM2-like). Moreover, the features of PMRlnc were upregulated in IM2/IM2-like tumors but downregulated in IM4/IM4-like tumors, suggesting their potential involvement in metabolic and immune regulation. The absence of significant survival differences between PMRlnc risk groups in the CheckMate cohort may reflect the extensive biological and microenvironmental remodeling induced by targeted and immune therapies, which could mask the intrinsic prognostic signals captured by the model. This finding suggested that PMRlnc may be more suitable for predicting natural disease progression prior to systemic treatment, while the derived classification could also provide insights to guide therapeutic decision-making.

This study has several limitations. Although our pipeline provided a systematic framework for inferring lncRNA functions, the proposed mechanisms remain preliminary and require more experimental validation. Second, although DMRlnc and PMRlnc demonstrated robust prognostic performance, an expansion to larger external cohorts will be necessary to strengthen statistical power and clinical relevance. Third, since DMRlnc and PMRlnc were derived from exploratory and retrospective subgroup analyses, their clinical utility should be validated in large-scale, multicenter, and prospective studies specifically designed for this purpose. In addition, DMRlnc and PMRlnc shown particularly strong performance in ccRCC and pRCC but not in chRCC, which may be attributable to differences in underlying cell types and could limit their generalizability.

## Conclusion

In conclusion, we systematically analyzed lncRNA characterization and clinical applications in ccRCC. We found that malignant cells exhibited broader but lower lncRNA expression, likely reflecting CNVs, whereas normal epithelial cells expressed low-abundance lncRNAs with higher individual variability. Our pipeline provided a framework for preliminary inference of lncRNA functions and identified five lncRNAs associated with metabolic and immune pathways. Importantly, we developed and independently validated two concise lncRNA based models for ccRCC diagnosis and prognosis. Both models demonstrated stable performance across multiple external cohorts, effectively distinguishing tumor from normal tissues and stratifying patients into clinically meaningful risk groups, which supported refined risk assessment, prognostic evaluation, and personalized therapeutic decision-making. Collectively, these findings provided a foundation for future mechanistic studies of lncRNAs and highlighted their translational potential in the clinical management of ccRCC.

## Supplementary Material

Supplementary figures.

Supplementary table 1.

Supplementary table 2.

Supplementary table 3.

Supplementary table 4.

## Data Availability

Transcriptome and metabolome datasets generated during the current study are available upon reasonable request. Raw sequencing data have been uploaded to the GSA-Human database under accession code PRJCA014547 (https://ngdc.cncb.ac.cn/bioproject/browse/PRJCA014547), but a DAC (discretionary access control) approval is necessary due to policy restrictions. Every researcher could submit an application on the website, and it would commonly take several weeks for the database administrator and DAC to review. All the processed sequencing data have been uploaded to Zenodo (https://zenodo.org/record/8063124) and figshare100 (https://doi.org/10.6084/ m9.figshare.24599295). Expression matrix of TCGA KIRC along with clinical features was obtained from UCSC Xena (https://xenabrowser.net/datapages/?cohort=GDC%20TCGA%20Kidney%20Clear%20Cell%20Carcinoma%20(KIRC)&removeHub=https%3A%2F%2Fxena.treehouse.gi.ucsc.edu%3A443). JAVLIN and CheckMate datasets were obtained from the supplementary material of the original papers. Data of IMMOTION151 was obtained from the EGA (European Genome-Phenome Archive) database (https://ega-archive.org/studies/EGAS00001004353) with approval from the DAC.

## Abbreviations

ccRCC: clean cell renal cell carcinoma
RCC: renal cell carcinoma
ITH: intratumoral heterogeneity
LncRNAs: long non coding RNAs
DMRlnc: diagnosis model of ccRCC based on lncRNA
PMRlnc: prognosis model of ccRCC based on lncRNA
NAT: normal adjacent tissues
TCGA-KIRC: cancer genome atlas-kidney renal clear cell carcinoma
ANOVA: analysis of variance
snRNA-seq: single-nucleic RNA sequencing
bulk-seq: RNA sequencing
UMI: unique molecular identifier
PCA: principal components analysis
PT: proximal tubule
CNVs: copy number variations
hdWGCNA: high dimensional weighted gene co-expression network analysis
UMAP: uniform manifold approximation and projection
TPM: transcripts per million
kME: eigengene-based connectivity
LC/GC-MS: liquid chromatography/gas chromatography-mass spectrometry
DElncRNAs: differentially expressed lncRNAs
DElncRNAs (set1): DElncRNAs between 100 ccRCC tissues and 50 matched NATs
DElncRNAs (set2): DElncRNAs between the four subtypes of ccRCC
DEA: differential expression analysis
log2(FC): log2(fold change)
KEGG: kyoto encyclopedia of genes and genomes
CDF: cumulative distribution function
NMF: non-negative matrix factorization
RF-RFE: random forest recursive feature elimination
ROC: receiver operating characteristic
AUC: area under the ROC
GSEA: gene set enrichment analysis
GO: gene ontology
EVs: extracellular vehicles
PFS: progression-free survival
OS: overall survival
VEGFR: vascular endothelial growth factor receptor
DAC: discretionary access control
