# Supplementary material for: Multi-Omics Profiling of Long Noncoding RNAs in Clear Cell Renal Cell Carcinoma for Characterization and Clinical Applications
Source: Int J Biol Sci. 2026 Mar 25;22(7):3682–700. doi: 10.7150/ijbs.127291 (PMC13086019; doi:10.7150/ijbs.127291)
Supplement: Supplementary file 1 — Supplementary figures. [file ijbsv22p3682s1.pdf]

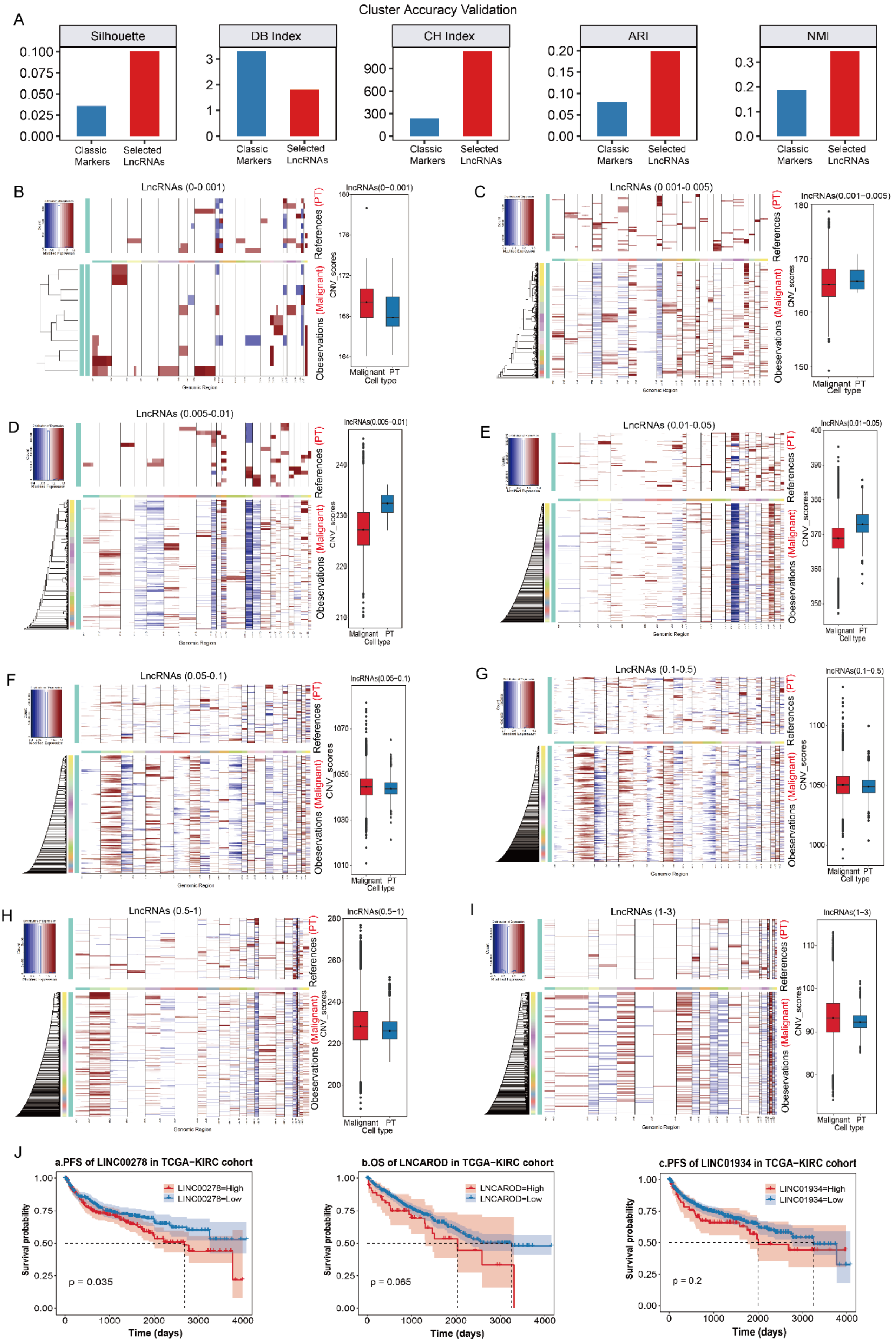

Cluster Accuracy Validation
A
Silhouette
DB Index
CH Index
ARI
NMI
Classic Markers
Selected LncRNAs
B
LncRNAs (0-0.001)
C
LncRNAs (0.001-0.005)
D
LncRNAs (0.005-0.01)
E
LncRNAs (0.01-0.05)
F
LncRNAs (0.05-0.1)
G
LncRNAs (0.1-0.5)
H
LncRNAs (0.5-1)
I
LncRNAs (1-3)
References (PT)
Obeservations (Malignant)
Genomic Region
CNV_scores
Malignant PT
Cell type
J
a.PFS of LINC00278 in TCGA−KIRC cohort
LINC00278=High
LINC00278=Low
p = 0.035
b.OS of LNCAROD in TCGA−KIRC cohort
LNCAROD=High
LNCAROD=Low
p = 0.065
c.PFS of LINC01934 in TCGA−KIRC cohort
LINC01934=High
LINC01934=Low
p = 0.2
Survival probability
Time (days)

SFig. 1 | CNV heatmap and CNV score of lncRNAs with different range

(A) Internal clustering metrics (Silhouette coefficient, CH index, and DB index) and external metrics (ARI and NMI) comparing classic protein-coding markers with selected lncRNAs with specific expression patterns. **(B-H)** CNV heatmap and CNV scores of lncRNAs across different ranges: **(B)** 0-0.001, **(C)** 0.001-0.005, **(D)** 0.005-0.01, **(E)** 0.01-0.05, **(F)** 0.05-0.1, **(G)** 0.1-0.5, **(H)** 0.5-1, and (H) 1-3. **(J)** Survival analysis of LINC00278 **(a)**, LNCAROD **(b)** and LINC01934 **(c)** in the TCGA-KIRC cohort.

Abbreviations: CH index, Calinski-Harabasz index; DB index, Davies-Bouldin index; ARI, adjusted rand index; NMI, normalized mutual information; CNV, copy number variations; OS, overall survival; PFS: progression-free survival.

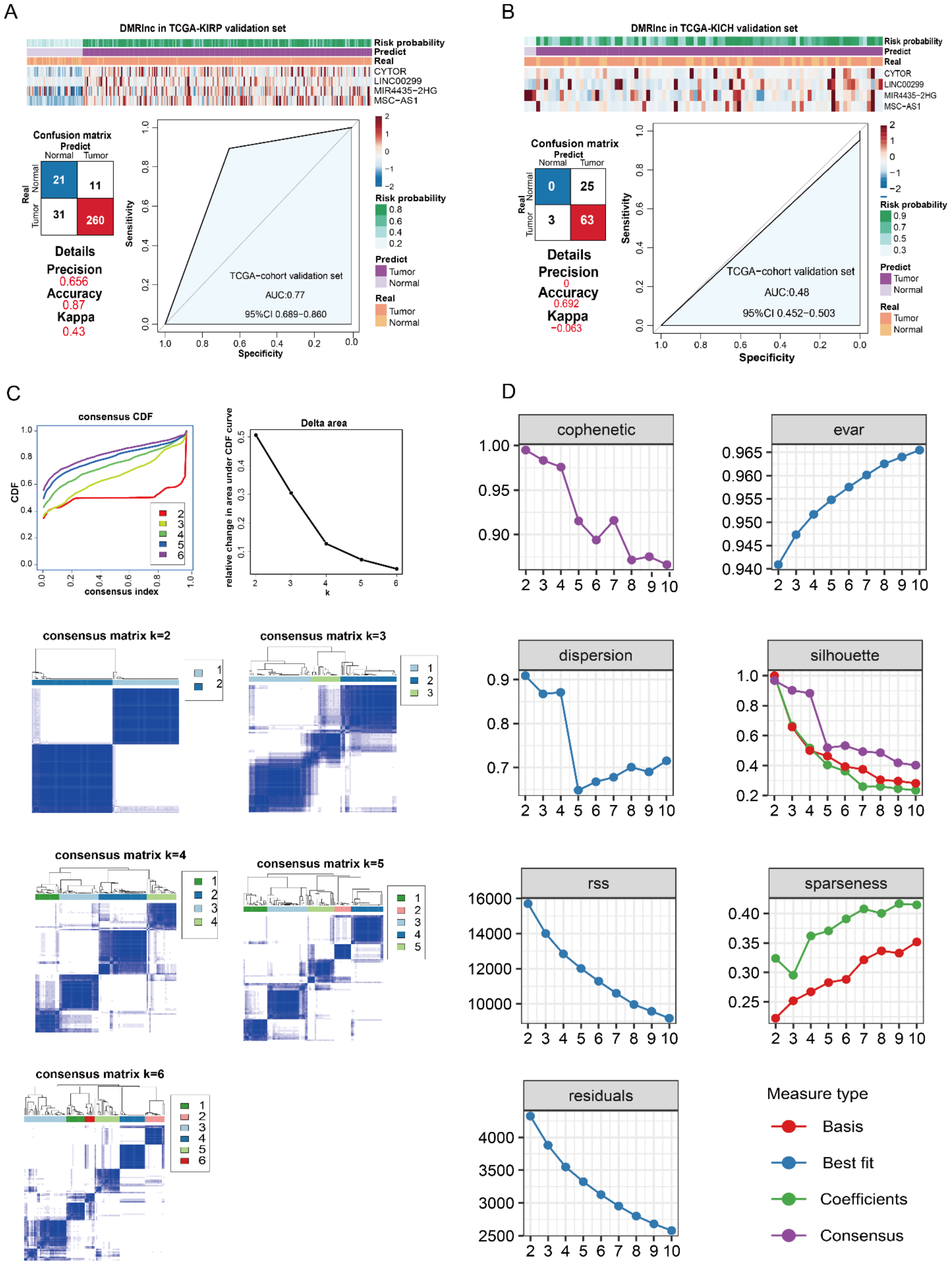
A
DMRlnc in TCGA-KIRP validation set
Risk probability
Predict
Real
CYTOR
LINC00299
MIR4435−2HG
MSC−AS1
Confusion matrix
Predict
Normal
Tumor
Real
21
11
31
260
Details
Precision
0.656
Accuracy
0.87
Kappa
0.43
Sensitivity
Specificity
TCGA−cohort validation set
AUC:0.77
95%CI 0.689−0.860
B
DMRlnc in TCGA-KICH validation set
0
25
3
63
Precision
0
Accuracy
0.692
Kappa
−0.063
TCGA−cohort validation set
AUC:0.48
95%CI 0.452−0.503
C
consensus CDF
CDF
consensus index
Delta area
relative change in area under CDF curve
k
consensus matrix k=2
consensus matrix k=3
consensus matrix k=4
consensus matrix k=5
consensus matrix k=6
D
cophenetic
evar
dispersion
silhouette
rss
sparseness
residuals
Measure type
Basis
Best fit
Coefficients
Consensus

SFig. 2 | Validation of DMRlnc in other kidney cancers and clusters based on lncRNA markers

(A) Heatmap, confusion matrix, and ROC curve showing the expression patterns and consistency of DMRlnc in the TCGA-KIRP validation cohort. **(B)** Heatmap, confusion matrix, and ROC curve showing the expression patterns and consistency of DMRlnc in the TCGA-KICH validation cohort. **(C)** Consensus CDF and delta area curves identifying k = 4 as the optimal clustering solution, with consensus matrices shown for k = 2-6. **(D)** NMF rank survey confirming k = 4 as the optimal rank.

Abbreviations: CDF, cumulative distribution function; NMF, non-negative matrix factorization.

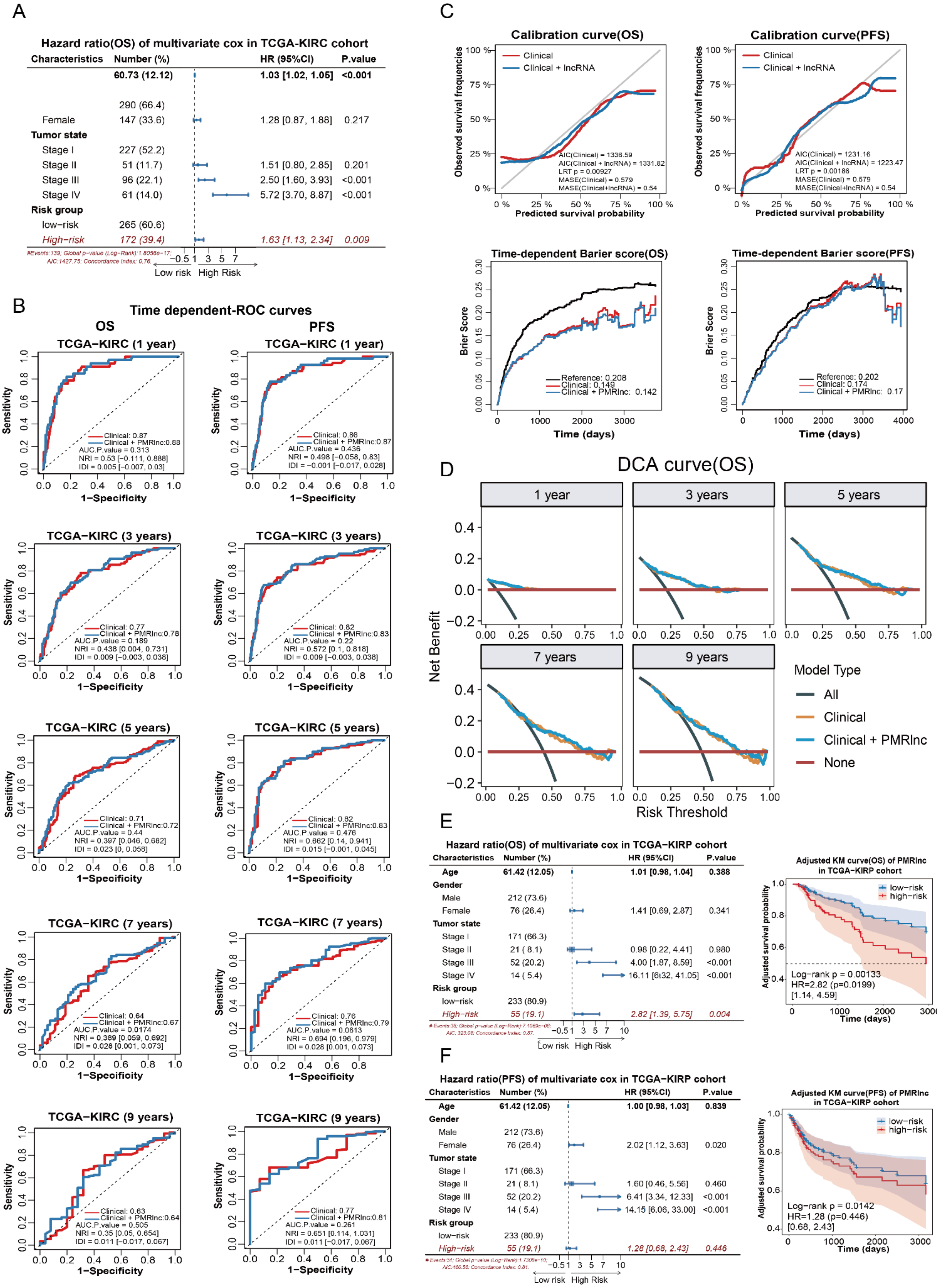
A
Hazard ratio(OS) of multivariate cox in TCGA-KIRC cohort
Characteristics Number (%) HR (95%CI) P.value
60.73 (12.12) 1.03 [1.02, 1.05] <0.001
290 (66.4)
Female 147 (33.6) 1.28 [0.87, 1.88] 0.217
Tumor state
Stage I 227 (52.2)
Stage II 51 (11.7) 1.51 [0.80, 2.85] 0.201
Stage III 96 (22.1) 2.50 [1.60, 3.93] <0.001
Stage IV 61 (14.0) 5.72 [3.70, 8.87] <0.001
Risk group
low−risk 265 (60.6)
High−risk 172 (39.4) 1.63 [1.13, 2.34] 0.009
Low risk High Risk
B
Time dependent-ROC curves
OS
PFS
TCGA−KIRC (1 year)
TCGA−KIRC (3 years)
TCGA−KIRC (5 years)
TCGA−KIRC (7 years)
TCGA−KIRC (9 years)
Sensitivity
1−Specificity
C
Calibration curve(OS)
Calibration curve(PFS)
Clinical
Clinical + lncRNA
Observed survival frequencies
Predicted survival probability
AIC(Clinical) = 1336.59
AIC(Clinical + lncRNA) = 1331.82
LRT p = 0.00927
MASE(Clinical) = 0.579
MASE(Clinical+lncRNA) = 0.54
AIC(Clinical) = 1231.16
AIC(Clinical + lncRNA) = 1223.47
LRT p = 0.00186
MASE(Clinical) = 0.579
MASE(Clinical+lncRNA) = 0.54
Time-dependent Barier score(OS)
Time-dependent Barier score(PFS)
Brier Score
Time (days)
Reference: 0.208
Clinical: 0.149
Clinical + PMRlnc: 0.142
Reference: 0.202
Clinical: 0.174
Clinical + PMRlnc: 0.17
D
DCA curve(OS)
1 year
3 years
5 years
7 years
9 years
Net Benefit
Risk Threshold
Model Type
All
Clinical
Clinical + PMRlnc
None
E
Hazard ratio(OS) of multivariate cox in TCGA-KIRP cohort
Characteristics Number (%) HR (95%CI) P.value
Age 61.42 (12.05) 1.01 [0.98, 1.04] 0.388
Gender
Male 212 (73.6)
Female 76 (26.4) 1.41 [0.69, 2.87] 0.341
Tumor state
Stage I 171 (66.3)
Stage II 21 (8.1) 0.98 [0.22, 4.41] 0.980
Stage III 52 (20.2) 4.00 [1.87, 8.59] <0.001
Stage IV 14 (5.4) 16.11 [6.32, 41.05] <0.001
Risk group
low−risk 233 (80.9)
High−risk 55 (19.1) 2.82 [1.39, 5.75] 0.004
Low risk High Risk
Adjusted KM curve(OS) of PMRlnc in TCGA−KIRP cohort
low−risk
high−risk
Adjusted survival probability
Log−rank p = 0.00133
HR=2.82 (p=0.0199)
[1.14, 4.59]
Time (days)
F
Hazard ratio(PFS) of multivariate cox in TCGA-KIRP cohort
Characteristics Number (%) HR (95%CI) P.value
Age 61.42 (12.05) 1.00 [0.98, 1.03] 0.839
Gender
Male 212 (73.6)
Female 76 (26.4) 2.02 [1.12, 3.63] 0.020
Tumor state
Stage I 171 (66.3)
Stage II 21 (8.1) 1.60 [0.46, 5.56] 0.460
Stage III 52 (20.2) 6.41 [3.34, 12.33] <0.001
Stage IV 14 (5.4) 14.15 [6.06, 33.00] <0.001
Risk group
low−risk 233 (80.9)
High−risk 55 (19.1) 1.28 [0.68, 2.43] 0.446
Low risk High Risk
Adjusted KM curve(PFS) of PMRlnc in TCGA−KIRP cohort
low−risk
high−risk
Adjusted survival probability
Log−rank p = 0.0142
HR=1.28 (p=0.446)
[0.68, 2.43]
Time (days)

SFig. 3 | Comparison between PMRlnc and clinical variables

(A) Forest plot of HR for OS derived from multivariate cox regression including PMRlnc and clinical variables in the TCGA-KIRC cohort. **(B)** Time-dependent ROC curves and NRI/IDI analyses comparing PMRlnc alone and PMRlnc combined with clinical variables at 1, 3, 5, 7, and 9 years in the TCGA-KIRC cohort. **(C)** Calibration curves and time-dependent barrier scores for PMRlnc and the combined PMRlnc + clinical model for OS and PFS in the TCGA-KIRC cohort; closer alignment to the diagonal and lower barrier scores indicated better calibration. **(D)** DCA comparing the clinical model alone with the combined clinical + PMRlnc model for OFS prediction. **(E)** Forest plot of HR for OS from multivariate Cox regression including PMRlnc and clinical variables, together with adjusted Kaplan-Meier curves, in the TCGA-KIRP cohort. **(F)** Forest plot of HR for OS from multivariate Cox regression including PMRlnc and clinical variables, together with adjusted Kaplan-Meier curves, in the TCGA-KIRP cohort.

Abbreviations: NRI, net reclassification improvement; IDI, integrated discrimination improvement; DCA, decision curve analysis; HR, hazard ratios.

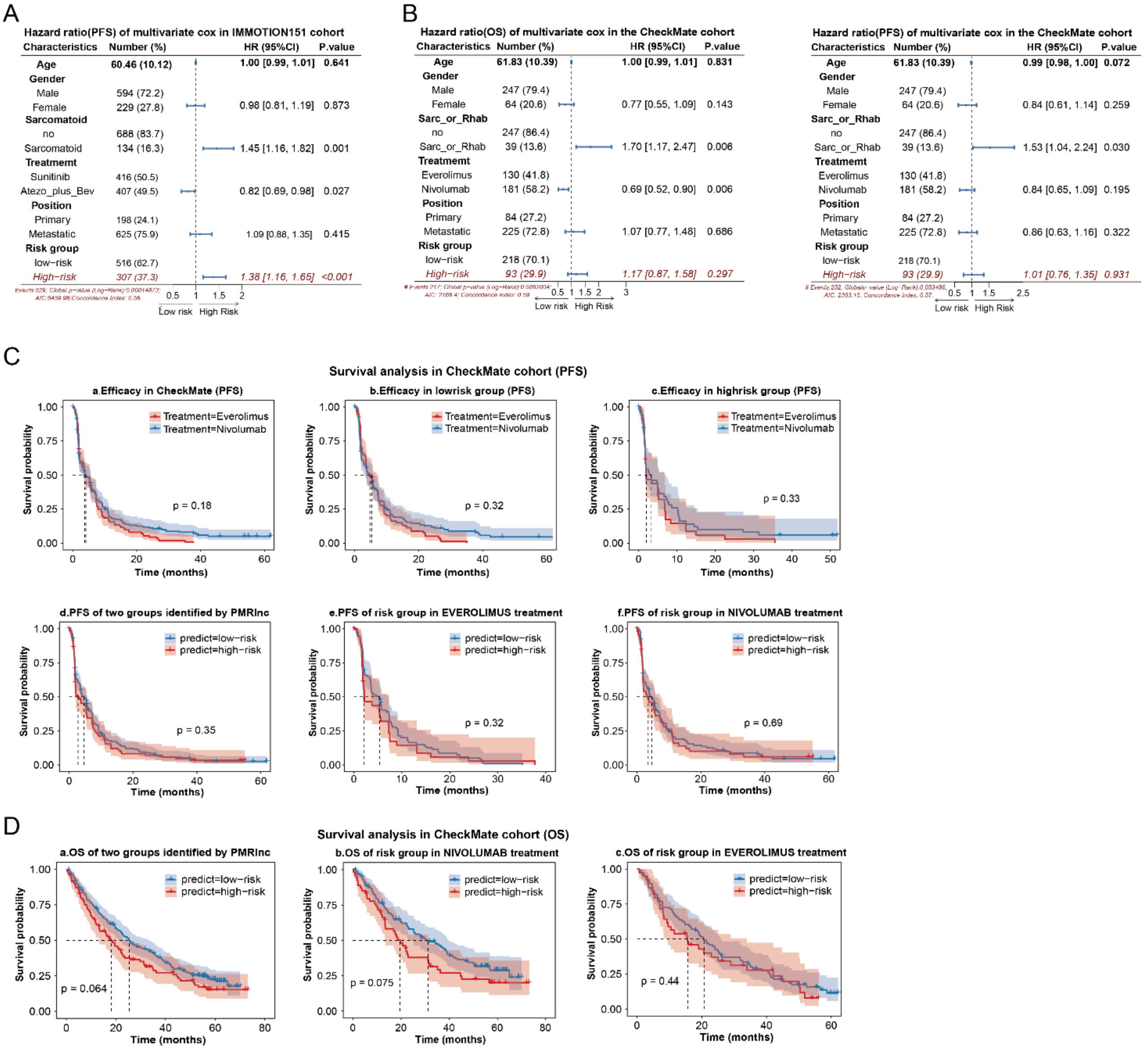
A
Hazard ratio(PFS) of multivariate cox in IMMOTION151 cohort
Characteristics Number (%) HR (95%CI) P.value
Age 60.46 (10.12) 1.00 [0.99, 1.01] 0.641
Gender
Male 594 (72.2)
Female 229 (27.8) 0.98 [0.81, 1.19] 0.873
Sarcomatoid
no 688 (83.7)
Sarcomatoid 134 (16.3) 1.45 [1.16, 1.82] 0.001
Treatmemt
Sunitinib 416 (50.5)
Atezo_plus_Bev 407 (49.5) 0.82 [0.69, 0.98] 0.027
Position
Primary 198 (24.1)
Metastatic 625 (75.9) 1.09 [0.88, 1.35] 0.415
Risk group
low−risk 516 (62.7)
High−risk 307 (37.3) 1.38 [1.16, 1.65] <0.001
Low risk High Risk
B
Hazard ratio(OS) of multivariate cox in the CheckMate cohort
Characteristics Number (%) HR (95%CI) P.value
Age 61.83 (10.39) 1.00 [0.99, 1.01] 0.831
Gender
Male 247 (79.4)
Female 64 (20.6) 0.77 [0.55, 1.09] 0.143
Sarc_or_Rhab
no 247 (86.4)
Sarc_or_Rhab 39 (13.6) 1.70 [1.17, 2.47] 0.006
Treatmemt
Everolimus 130 (41.8)
Nivolumab 181 (58.2) 0.69 [0.52, 0.90] 0.006
Position
Primary 84 (27.2)
Metastatic 225 (72.8) 1.07 [0.77, 1.48] 0.686
Risk group
low−risk 218 (70.1)
High−risk 93 (29.9) 1.17 [0.87, 1.58] 0.297
Low risk High Risk
Hazard ratio(PFS) of multivariate cox in the CheckMate cohort
Characteristics Number (%) HR (95%CI) P.value
Age 61.83 (10.39) 0.99 [0.98, 1.00] 0.072
Gender
Male 247 (79.4)
Female 64 (20.6) 0.84 [0.61, 1.14] 0.259
Sarc_or_Rhab
no 247 (86.4)
Sarc_or_Rhab 39 (13.6) 1.53 [1.04, 2.24] 0.030
Treatmemt
Everolimus 130 (41.8)
Nivolumab 181 (58.2) 0.84 [0.65, 1.09] 0.195
Position
Primary 84 (27.2)
Metastatic 225 (72.8) 0.86 [0.63, 1.16] 0.322
Risk group
low−risk 218 (70.1)
High−risk 93 (29.9) 1.01 [0.76, 1.35] 0.931
Low risk High Risk
C
Survival analysis in CheckMate cohort (PFS)
a.Efficacy in CheckMate (PFS)
Treatment=Everolimus
Treatment=Nivolumab
p = 0.18
b.Efficacy in lowrisk group (PFS)
p = 0.32
c.Efficacy in highrisk group (PFS)
p = 0.33
d.PFS of two groups identified by PMRlnc
predict=low−risk
predict=high−risk
p = 0.35
e.PFS of risk group in EVEROLIMUS treatment
p = 0.32
f.PFS of risk group in NIVOLUMAB treatment
p = 0.69
Survival probability
Time (months)
D
Survival analysis in CheckMate cohort (OS)
a.OS of two groups identified by PMRlnc
p = 0.064
b.OS of risk group in NIVOLUMAB treatment
p = 0.075
c.OS of risk group in EVEROLIMUS treatment
p = 0.44
predict=low−risk
predict=high−risk
Survival probability
Time (months)

SFig. 4 | Survival analysis in IMMOTION151 and CheckMate cohort

(A) Forest plot of HR for PFS derived from multivariate cox regression including PMRlnc and clinical variables in the IMMMTION151 cohort. **(B)** Forest plot of HR for OS and PFS derived from multivariate cox regression including PMRlnc and clinical variables in the CheckMate cohort. **(C) a-c:** PFS of treatment groups in the Checkmate cohort(a); the low-risk group(b); the high-risk group(c). **d-f:** PFS comparison of high risk and low risk groups (everolimus vs. nivolumab) in the entire Checkmate cohort(d), the everolimus group(e), and the nivolumab group(f). **(D)** OS comparison of high risk and low risk groups (everolimus vs. nivolumab) in the entire Checkmate cohort(a), the everolimus group(b), and the nivolumab group(c).

Abbreviations: OS, overall survival; PFS: progression-free survival.
